# Reflux symptoms and oesophageal acidification in treated achalasia patients are often not reflux related

**DOI:** 10.1136/gutjnl-2020-320772

**Published:** 2020-05-21

**Authors:** Fraukje A Ponds, Jacobus M Oors, André J P M Smout, Albert J Bredenoord

**Affiliations:** Gastroenterology and Hepatology, Amsterdam UMC, University of Amsterdam, Amsterdam, The Netherlands

**Keywords:** achalasia, oesophageal ph monitoring, visceral hypersensitivity, oesophageal motility disorder, gastroesophageal reflux disease

## Abstract

**Objective:**

After treatment, achalasia patients often develop reflux symptoms. Aim of this case–control study was to investigate mechanisms underlying reflux symptoms in treated achalasia patients by analysing oesophageal function, acidification patterns and symptom perception.

**Design:**

Forty treated achalasia patients (mean age 52.9 years; 27 (68%) men) were included, 20 patients with reflux symptoms (RS+; Gastro-Oesophageal Reflux Disease Questionnaire (GORDQ) ≥8) and 20 without reflux symptoms (RS−: GORDQ <8). Patients underwent measurements of oesophagogastric junction distensibility, high-resolution manometry, timed barium oesophagogram, 24 hours pH-impedance monitoring off acid-suppression and oesophageal perception for acid perfusion and distension. Presence of oesophagitis was assessed endoscopically.

**Results:**

Total acid exposure time during 24 hours pH-impedance was not significantly different between patients with (RS+) and without (RS−) reflux symptoms. In RS+ patients, acid fermentation was higher than in RS− patients (RS+: mean 6.6% (95% CI 2.96% to 10.2%) vs RS−: 1.8% (95% CI −0.45% to 4.1%, p=0.03) as well as acid reflux with delayed clearance (RS+: 6% (95% CI 0.94% to 11%) vs RS−: 3.4% (95% CI −0.34% to 7.18%), p=0.051). Reflux symptoms were not related to acid in both groups, reflected by a low Symptom Index. RS+ patients were highly hypersensitive to acid, with a much shorter time to heartburn perception (RS+: 4 (2–6) vs RS−:30 (14-30) min, p<0.001) and a much higher symptom intensity (RS+: 7 (4.8–9) vs RS−: 0.5 (0–4.5) Visual Analogue Scale, p<0.001) during acid perfusion. They also had a lower threshold for mechanical stimulation.

**Conclusion:**

Reflux symptoms in treated achalasia are rarely caused by gastro-oesophageal reflux and most instances of oesophageal acidification are not reflux related. Instead, achalasia patients with post-treatment reflux symptoms demonstrate oesophageal hypersensitivity to chemical and mechanical stimuli, which may determine symptom generation.

**Significance of this study**

**What is already known on this subject?**

- After treatment achalasia patients often develop reflux symptoms.
- These symptoms are considered to be related to gastro-oesophageal reflux.
- Treatment focus on acid suppression which has a variable efficacy on reducing symptoms.

**What are the new findings?**

- Reflux symptoms in treated achalasia patients are rarely caused by gastro-oesophageal reflux.
- Oesophageal acidification is frequently observed during pH-impedance monitoring after achalasia treatment which is partially reflux induced but largely due to acid fermentation or acidic food-induced stasis.
- Oesophageal hypersensitivity to chemical and mechanical stimuli seems to play a role in the generation of reflux symptoms in treated achalasia patients.

**How might it impact on clinical practice in the foreseeable future?**

- Diagnostic approach and management of treated achalasia patients with reflux-like symptoms should be altered.
- Treatment of these symptoms should not be limited to acid suppression, but targeted on reducing both oesophageal acidification and oesophageal hypersensitivity.

## Introduction

Achalasia and gastro-oesophageal reflux disease (GORD) represent opposite ends of the spectrum of oesophagogastric junction (OGJ) dysfunction. Achalasia is a rare oesophageal motility disorder characterised by absent peristalsis of the oesophageal body and impaired relaxation of the lower oesophageal sphincter (LOS), which hampers oesophageal emptying. GORD is one of most common gastrointestinal disorders worldwide and is the result of an unusually weak OGJ which induces retrograde flow of gastric content into the oesophagus resulting into troublesome symptoms and/or mucosal damage.^1^ Treatment of achalasia focuses on symptom relief achieved by disruption of the LOS, compromising the OGJ barrier against reflux. Post-treatment, the prevalence of reflux symptoms and/or reflux oesophagitis in achalasia patients varies between 5% and 60%.^2–6^ The variability in reflux prevalence is related to the definition and measurement of reflux and to the type of treatment, since there is a higher rate of reflux symptoms after laparoscopic or endoscopic myotomy without fundoplication compared with pneumodilation or myotomy with fundoplication.^2–9^ Studies in these patients showed that reflux symptoms, pH monitoring and/or reflux oesophagitis in post-treatment achalasia patients correlate poorly.^10–15^ True reflux as the cause of reflux symptoms was inconsistently observed. Nevertheless, it is common practice to consider reflux symptom of treated achalasia patients as GORD and start proton pump inhibitors (PPI), which has variable efficacy. The underlying mechanisms of these symptoms are thus poorly investigated, which hampers adequate and tailored treatment. Therefore, the aim of this study was to thoroughly investigate the mechanisms underlying reflux symptoms in treated achalasia by analysing oesophageal function, acid exposure, acidification patterns, symptom perception and reflux oesophagitis.

## Material and methods

### Study subjects and inclusion criteria

In this prospective observational case–control study, treated adult (≥18 years) achalasia patients visiting the outpatient clinic of the Gastroenterology and Hepatology Department of the Amsterdam UMC were approached to participate in the study. Patients were allocated into two groups depending on whether or not reflux symptoms were present. Treated achalasia patients with a total score of ≥8 on the Gastro-Oesophageal Reflux Disease Questionnaire (GORDQ) were classified as having reflux symptoms (RS+) and a score ≤8 as without reflux symptoms (RS−).^16 17^ The GORDQ is a widely used, validated, 6-item self-report questionnaire, evaluating reflux symptoms (heartburn, regurgitation and chest pain), sleep disturbance by reflux and antacid use, range per item 0–3 with a minimum total score of 0 and a maximum score of 18.^16 17^ The GORDQ was completed while the patients were off acid suppression. All included achalasia patients were ≥6 months post-treatment and in clinical remission for achalasia, defined as an Eckardt score ≤3. The Eckardt symptom score assesses the severity of achalasia symptoms by the sum of symptom frequency scores for dysphagia, regurgitation and chest pain (range 0–3: 0: absent; 1: occasionally; 2: daily; 3: every meal) combined with a weight loss score (range 0–3: 0: no weight loss; 1: <5 kg weight loss; 2: 5–10 kg weight loss; 3: >10 kg weight loss) resulting in a minimum score of 0 until a maximum score, indicating most pronounced symptoms, of 12.^18^ In all patients, the diagnosis of achalasia was previously confirmed by oesophageal manometry before treatment, showing absent peristalsis and impaired LOS relaxation. Treatment consisted of endoscopic pneumodilation, laparoscopic Heller’s myotomy with Dor fundoplication 180° and/or peroral endoscopic myotomy (POEM). Pneumodilations started with a 30 mm balloon, followed by a 35 mm balloon and in case of persistent symptoms a 40 mm balloon was used. Detailed eligibility criteria are provided in the study protocol (online supplementary 1 and 2).

10.1136/gutjnl-2020-320772.supp1 Supplementary data

The study was registered in the Dutch trial registry before the start of the study (NTR3838, trialregister.nl). Written informed consent was obtained from all patients before study participation. Normal values for acid sensitivity in healthy subjects were obtained in a previous study.^19^

### Study protocol

**Figure 1:**
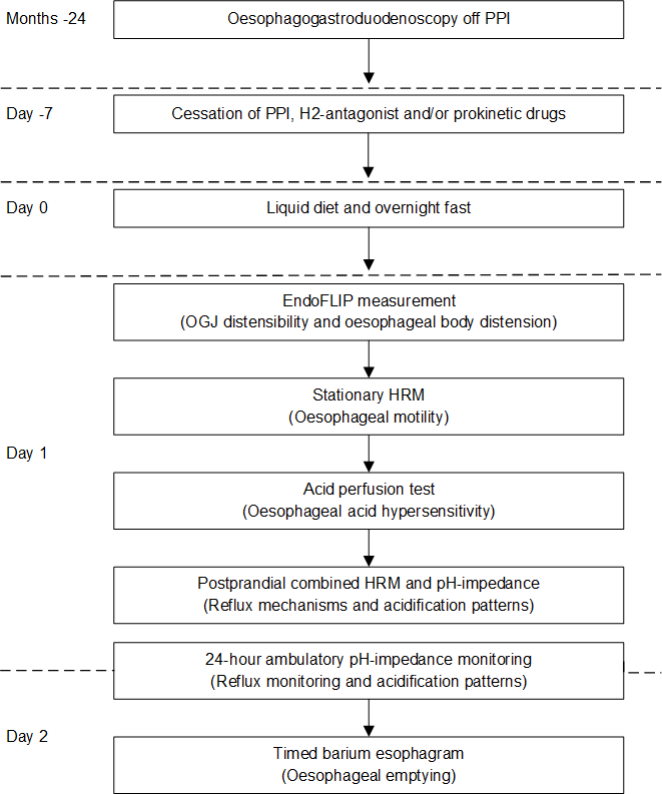
Study protocol. EndoFLIP, Endo Functional Luminal Imaging Probe; HRM, high-resolution manometry; OGJ, oesophagogastric junction; PPI, proton pump inhibitors.

Measurements were performed on two subsequent days after cessation of PPI, H2-receptor antagonists and/or prokinetic medication for 1 week (figure 1). Baseline data and questionnaires (Eckardt score, GORDQ, Reflux Disease Questionnaire (RDQ), Achalasia Disease-Specific Quality-of-Life questionnaire and Medical Outcomes Study 36-Item Short-Form Health Survey (SF-36)) were assessed before the measurements.^20 21^ One day before the measurements, patients were restricted to a liquid diet, followed by an overnight fast to minimise possible oesophageal stasis. Figure 1 displays the study protocol with the subsequent measurements that were performed during two study days including time intervals. On the first day, the distensibility of the OGJ was measured and oesophageal sensitivity for a mechanical stimulus was assessed (using EndoFLIP (Endo Functional Luminal Imaging Probe)), and stationary high-resolution manometry (HRM), acid perfusion test and a prolonged combined HRM and pH-impedance monitoring were performed. Thereafter the patients were dismissed, fitted with equipment for 24 hours ambulatory pH-impedance measurement. The next day, the 24 hours reflux monitoring was terminated and a timed barium oesophagogram was performed. Oesophagogastroduodenoscopy was not part of the protocol but in all patients performed off acid suppression as part of routine clinical practice before study participation. All measurements were analysed in a blinded fashion by the investigators.

### OGJ distensibility measurement and assessment of perception of oesophageal distension

To measure OGJ distensibility the EndoFLIP (Medtronic, Sunnyvale, California, USA) was used. By the use of impedance planimetry, EndoFLIP measures cross-sectional areas in the alimentary tract.^22^ Description of the EndoFLIP and the protocol to measure OGJ distensibility was previously described.^23^ The OGJ distensibility was determined at the 50 mL volume by dividing the median minimal cross-sectional area, reflecting the OGJ, by the median intrabag pressure during the complete recording period, expressed as mm^2^/mm Hg.

After measuring OGJ distensibility, the catheter was placed 10 cm above the OGJ to evaluate perception of oesophageal distension. The EndoFLIP bag was inflated from 20 to 70 mL, with a 10 mL increase of volume each minute. After each inflation, patients were asked to notify their first perception of the mechanical stimulus and to score the intensity of their perception on a Visual Analogue Scale (VAS), a horizontal 100 mm line marked with ‘no pain’ and ‘most extreme pain’.

### High-resolution manometry

HRM was performed using a 22-channel water-perfused catheter (Laborie, Williston, Vermont, USA) with an incorporated infusion channel until the end of the catheter. The proximal, first 15 channels were spaced at 2 cm intervals, followed by 6 channels at 1 cm intervals for measuring the LOS and the last channel at the end of the catheter to measure gastric pressure. The catheter was introduced transnasally and positioned to measure from hypopharynx to stomach. Following a standardised protocol, patients were placed in supine position (20°) and received 10 boluses of 5 mL water with an interval of 20 s. Prior and subsequently to the swallows, a period of 30 s not swallowing was assessed for baseline measures. Manometric signals were recorded with a frequency of 20 Hz. The HRM studies were analysed by dedicated software (Laborie, Williston, Vermont, USA), according to the Chicago classification V.3, adjusted for water-perfused values.^24 25^ The following key oesophageal pressure topography metrics were assessed: OGJ basal pressure at end-expiration, the 4 s integrated relaxation pressure (IRP), distal contractile integral, distal latency, peristaltic integrity using the 20 mm Hg isobaric contour and intrabolus pressure pattern with ≥30 mm Hg isobaric contour.^24 26^ OGJ/LOS pressure was referenced to gastric pressure and oesophageal contraction metrics to atmospheric pressure.^24 26^

### Acid perfusion test

An acid perfusion test was performed according to a previously described protocol.^19^ The water-perfused HRM catheter was used with the incorporated infusion channel 6 cm above the OGJ. Patients were in semirecumbent position. After an adaptation period of 10 min, perfusion with a neutral solution (saline, NaCl 0.9% at pH 6.5) was performed for 10 min, followed by an acidic solution (0.1 n HCl at pH 1.1) for 30 min. The perfusion rate was 8 mL/min. Patients were blinded for the nature of the solutions and unaware of the switch to acid perfusion. The time to first perception of heartburn and time to discomfort were noted. Symptom severity was scored every 2 min on a VAS, a horizontal 100 mm line marked with ‘no pain’ and ‘most extreme pain’ labelled at the beginning and end of the line. Perfusion was stopped when symptoms were intolerable. A perfusion sensitivity score was calculated as follows: ((total perfusion time – lag time to perception) x maximum VAS), conform previously described methods.^19 27^ Patients with a first perception of heartburn within 20 min after acid perfusion were considered increased hypersensitive to acid.^19^

### Postprandial stationary HRM and pH-impedance measurement

Combined HRM and pH-impedance monitoring was performed after the acid perfusion test. The pH-impedance catheter consisted of 6 impedance segments and 1 ISFET pH electrode (Unisensor AG, Attikon, Switzerland) and was placed next to the HRM catheter, with the pH electrode 5 cm above the upper border of the LOS. The impedance segments were located at 2–4, 4–6, 6–8, 8–10, 14–16 and 16–18 cm above the upper border of the LOS. A second pH catheter without impedance electrodes was placed 10 cm above the upper border of the LOS to detect the proximal extent of reflux or acidification beside the impedance measurement. Low distal baseline impedance tracings could prevent adequate detection of proximal acid exposure. Impedance (50 Hz), pH and pressure (20 Hz) signals were recorded and stored on a computer with dedicated software (Laborie, Williston, Vermont, USA). After an adaptation period of 30 min, intragastric infusion of a standardised high-caloric liquid meal, 250 mL nutrient drink (600 kcal, 18 g protein, Nutridrink Compact Protein, Nutricia, Zoetermeer, The Netherlands) diluted by 150 mL water, was started. For the perfusion the incorporated infusion channel of the HRM catheter was used with a perfusion rate of 13 mL/min during 30 min. This was followed by a postprandial measurement during 120 min. Impedance and pH tracings were analysed for acid patterns (see definitions below), acid exposure time (percentage of time pH <4), occurrence of reflux episodes according to previously described criteria and discriminating reflux from fermentation.^28 29^ Combined HRM and pH-impedance monitoring was used to detect mechanisms of acid exposure (eg, swallow induced, transient LOS relaxation) and clearance.

### Twenty-four-hour ambulatory pH-impedance monitoring

**Figure 2:**
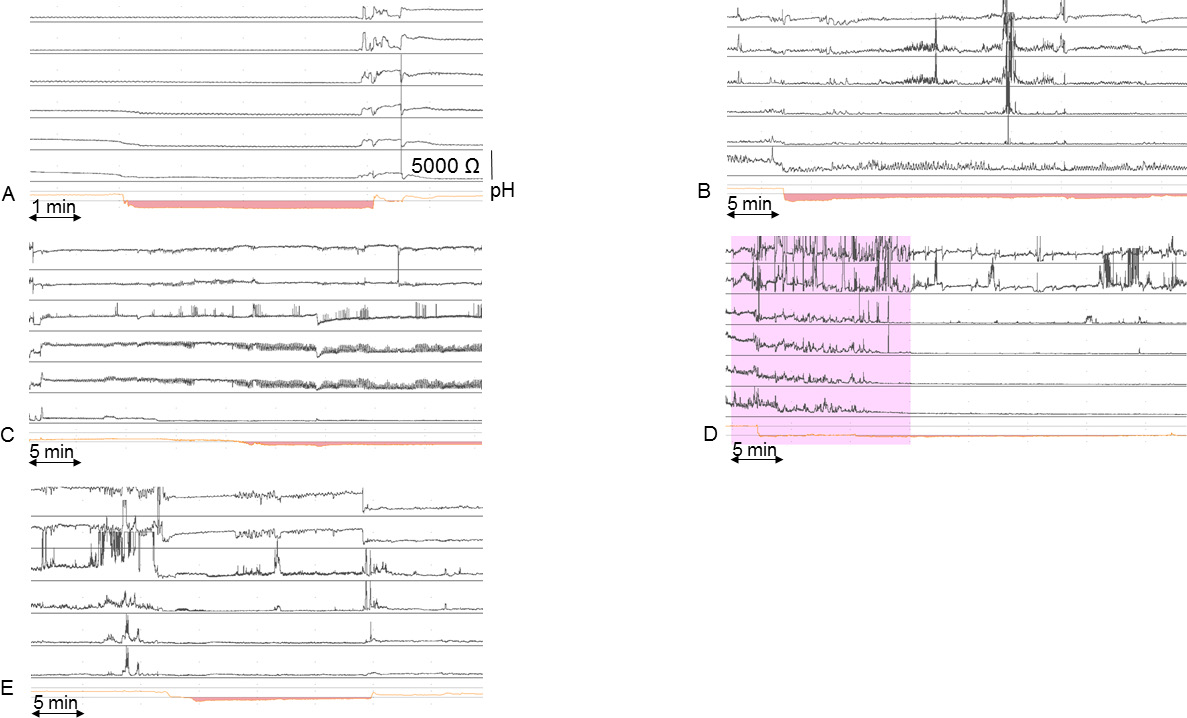
Oesophageal acidification patterns observed during pH-impedance monitoring. (A) Acid reflux with normal clearance: rapid pH drop to below 4, drop rate ≥1 pH unit per second, lasting between 10 s and 5 min. (B) Acid reflux with delayed clearance: rapid pH drop to below 4, drop rate ≥1 pH unit per second, lasting longer than 5 min. (C) Acid fermentation: slow pH drop to below 4, drop rate <1 pH unit per minute, lasting longer than 5 min. (D) Stasis of recently ingested acidic food or drink: pH drop to below 4 during meal/drink, pH below 4 persisting longer than 5 min after meal/drink. The pink-coloured area indicates meal/drink ingestion. (E) Unclassified: pH drop to below 4 not meeting criteria for any of the acid patterns described above.

After the combined HRM and pH-impedance monitoring, the HRM and single pH catheter were removed. The pH-impedance catheter stayed in place and was used for a 24-hour ambulatory measurement. The catheter was connected to a digital data logger (Laborie, Williston, Vermont, USA) to store pH and impedance signals at a frequency of 50 Hz. During the measurement, patients were instructed to consume meals and drinks at fixed times during the day and report symptoms in a diary. Analysing the ambulatory pH-impedance measurements, we distinguished five different acidification patterns: (A) acid reflux with normal clearance: rapid pH drop to below 4, drop rate ≥1 pH unit per second, lasting between 10 s and 5 minutes^28 29^; (B) acid reflux with delayed clearance: rapid pH drop to below 4, drop rate ≥1 pH unit per second, lasting longer than 5 min; (C) acid fermentation: slow pH drop to below 4, drop rate <1 pH unit per minute, lasting longer than 5 min; (D) stasis of recently ingested acidic food or drink: pH drop to below 4 during meal/drink, persisting longer than 5 min after meal/drink; (E) unclassified: pH drop to below 4 not meeting criteria for any of the acid patterns described above (figure 2). Low distal baseline impedance levels prevented the use of impedance for defining the observed acidification patterns. Impedance was used to identify prolonged acidification by further decrease of impedance levels (distal and proximal), clearance of acidification and air trapping. All acid episodes (pH <4) were analysed according to the predefined acidification patterns. Total acid exposure time, percentage of time pH <4, during the complete measurement and in upright and supine position were assessed. An acid exposure time >6% was considered pathological.^29^ The correlation between symptoms and acid patterns was analysed, with a positive correlation when symptoms were notified within 2 min from the start of the acid pattern. The Symptom Index (SI) was calculated by the number of symptoms associated with reflux as a percentage of the total number of symptoms. The optimal SI threshold was set at ≥50% of reported reflux symptoms.^27^ The symptom association probability was not calculated because in patients with achalasia the number of total acid reflux episodes cannot be determined reliably. Baseline impedance levels were measured every 2 hours in the proximal channel at 17 cm above the LOS and in the most distal channel at 3 cm above the LOS, as previously described.^30^ A 30 s time window was selected to calculate the baseline impedance by averaging the raw impedance values during this time period. The median values for proximal and distal impedance levels were calculated for the 24-hour measurement based on the 2 hours data.

### Timed barium oesophagogram and oesophagogastroduodenoscopy

A timed barium oesophagogram was performed on the second day, after the 24-hour pH-impedance monitoring. Patients were instructed to ingest a maximal tolerable amount of low density barium sulphate suspension up to 200 mL within 30–60 s in an upright, slight left posterior oblique position.^31^ Radiographs were taken at 0, 1, 2 and 5 min after ingestion of the suspension.^31^ To evaluate oesophageal emptying the barium column height at 5 min was measured from the OGJ to the top of the barium column in centimetres. Adequate oesophageal emptying was defined as ≤1 cm barium column height at 5 min. The maximal oesophageal diameter was measured by the oesophageal width at 5 min.

All patients had undergone an oesophagogastroduodenoscopy off PPI within 24 months before study participation. Severity of reflux oesophagitis was scored according to the Los Angeles classification.^32^

### Statistical analysis

An assumed 45% difference in objectified reflux episodes, between reflux symptomatic and asymptomatic treated achalasia patients, was hypothesised for the purpose of sample size calculations. With 19 patients in each group, the study would have 80% power to detect significant differences in outcome parameters that could give insight in the underlying mechanism of reflux symptoms. To compensate for technical failures, the aim was to enrol 20 patients per group, with a 1:1 allocation per group based on treatment type and gender.

Continuous data are presented as mean (SD or 95% CI) or median (IQR), according to distribution. Categorical data are presented as percentages. Continuous data were compared by unpaired Student’s t-test and one-way analysis of variance or Mann-Whitney U test and Kruskal-Wallis, according to distribution. Categorical data were analysed by χ^2^ and Fisher’s exact test. Relationships between parameters were analysed by linear regression analysis, (Pearson’s or Spearman’s correlation, r) or logistic regression analysis (OR with 95% CI). The time to perception during the acid perfusion test, was compared by the log rank test on Kaplan-Meier curves. A subgroup analysis of the outcome parameters of 24 hours pH-impedance, oesophagogastroduodenoscopy and provocation tests was also performed per treatment, laparoscopic Heller’s myotomy versus POEM. For both treatments, patients primarily treated by Heller’s myotomy or POEM were taken together with patients who failed on pneumodilation and retreated by Heller’s myotomy or POEM. Differences were considered statistically significant when p<0.05. All reported p values are two tailed. Statistical analysis was performed using IBM SPSS Statistics V.24 (IBM).

### Patient and public involvement

Patient involvement started at study inclusion. Patients assessed the burden of the study and gave feedback for adjustments on the study design for further studies. Patients were not involved in development of the research question, outcome measures or study design. Patients’ personal results of the measurements were shared on request. After publication, the article will be disseminated to all study participants and shared on the Dutch achalasia patient Facebook page.

## Results

### Patient characteristics

In total, 40 previously treated patients (mean age 52.9 years; 27 (68%) men) were included between March 2013 and December 2015, of whom 38 completed the study. Two patients failed to complete the study; one due to intolerance of the two catheters during prolonged stationary HRM/pH-impedance monitoring and the other because OGJ passage by HRM catheter did not succeed despite multiple attempts. The patient characteristics are shown in table 1. Age, body mass index, treatment type and Eckardt score were similar between the groups with (RS+) and without (RS−) reflux symptoms. The duration of PPI use since first prescription was significantly longer in patients with reflux symptoms, with a median period of 41 (19–82) months.

**Table 1:** Baseline characteristics of treated achalasia patients with (RS+) and without reflux symptoms (RS−)

	Patients with reflux symptoms (RS+) (n=19)	Patients without reflux symptoms (RS−) (n=19)	P value
Sex (n (%))			0.73
Male	11 (63)	12 (68)	
Female	8 (37)	7 (32)	
Age (years; mean (SD))	53.8 (13)	52.7 (13.5)	0.80
BMI (kg/m^2^; mean (SD))	25.7 (4.5)	25.7 (3.1)	0.94
Achalasia subtypes at diagnosis (n (%))			0.39
Type I	8 (42)	5 (26)	
Type II	10 (53)	13 (69)	
Type III	1 (5)	1 (5)	
Disease duration (years; (mean (SD))	7.8 (6.9)	8.3 (6.3)	0.81
Eckardt score (median (IQR))	2 (1–3)	2 (1–2)	0.43
GORDQ (median (IQR))*	11 (11–13)	6 (6–7)	<0.001
Achalasia treatment (n (%))			0.5
Pneumodilation†	1 (5)	1 (5)	
Laparoscopic Heller’s myotomy	5 (26)	4 (21)	
Peroral endoscopic myotomy	5 (26)	6 (32)	
Pneumodilation‡ and laparoscopic Heller’s myotomy	6 (32)	5 (26)	
Pneumodilation‡ and peroral endoscopic myotomy	2 (11)	3 (16)	
PPI use (n (%))	19 (100)	4 (21)	<0.001
Time PPI use post-treatment (months; (median (IQR))	41 (19–82)	0 (0–4)	<0.001

*GORD-Q: gastro-oesophageal reflux disease questionnaire, range 0–18, score ≥8 was highly suggestive for presence of GORD.

†Pneumodilation up till 35 mm balloon.

‡Pneumodilation up till 40 mm balloon.

BMI, body mass index; GORDQ, Gastro-Oesophageal Reflux Disease Questionnaire; PPI, proton pump inhibitor.

### Ambulatory 24-hour pH-impedance monitoring

An overview of the outcome parameters during the 24-hour ambulatory pH-impedance monitoring is shown in table 2. Surprisingly, no significant differences between the RS+ and RS− groups were observed in total, upright or supine acid exposure, nor in percentage of patients with pathological acid exposure. In 16% (3/19) of RS+ and in 26% (5/19) of RS− achalasia patients acid exposure was completely absent (p=0.43).

**Table 2:** Outcome of 24-hour pH-impedance monitoring of treated achalasia patients with (RS+) and without reflux symptoms (RS−)

	Patients with reflux symptoms (RS+)	Patients without reflux symptoms (RS−)	P value
	(n=19)	(n=19)	
24-hour pH-impedance monitoring			
Acid exposure time (AET: % of time pH <4; mean (95% CI))			
Total	13.8 (6.7 to 20.9)	10.9 (4.4 to 17.3)	0.53
Upright	10.9 (4.4 to 17.4)	6.6 (2.6 to 10.6)	0.24
Supine	17.7 (7.9 to 27.5)	16.4 (4.1 to 28.8)	0.86
Pathological acid exposure (AET pH <4 in >6%; n (%))	14 (74)	10 (53)	0.18
Acidification patterns (% of time; mean (95% CI))			
Acid reflux with normal clearance	0.2 (0.06 to 0.28)	0.09 (0.01 to 0.16)	0.39
Acid reflux with delayed clearance	6 (0.94 to 11.0)	3.4 (−0.34 to 7.18)	0.051
Acid fermentation	6.6 (2.96 to 10.2)	1.8 (−0.45 to 4.10)	0.03
Stasis of ingested acidic food	2.2 (−0.04 to 4.55)	7.6 (−0.13 to 15.3)	0.18
Unclassified	1.8 (−0.49 to 4.11)	0.01 (−0.02 to 0.04)	0.11
No of acidification events (median (IQR))			
Acid reflux with normal clearance	1 (0–2)	0 (0–1)	0.11
Acid reflux with delayed clearance	1 (0–3)	0 (0–1)	0.07
Acid fermentation	1 (0–3)	0 (0–0)	0.002
Stasis of ingested acidic food	0 (0–1)	0 (0–2)	0.54
Unclassified	0 (0–1)	0 (0–0)	0.008
No of patients per acidification pattern (n (%))			
Acid reflux with normal clearance	10 (53)	5 (26)	0.1
Acid reflux with delayed clearance	12 (63)	6 (32)	0.051
Acid fermentation	13 (68)	4 (21)	0.004
Stasis of ingested acidic food	6 (32)	8 (42)	0.5
Unclassified	8 (42)	2 (11)	0.03
No of symptoms (median (IQR))	4 (3–5)	0 (0–2)	<0.001
Baseline impedance (Ω; median (IQR))			
Proximal	2327 (1853–2836)	2638 (1659–3108)	0.93
Distal	487 (69–696)	476 (338–741)	0.84

AET, acid exposure time.

Episodes of acid reflux with normal clearance were rare in both groups (RS+: median 1 (0–2) vs RS−: 0 (0–1), p=0.11; table 2). RS+ achalasia patients had significantly more episodes of acid fermentation and unclassified acidification compared with RS− achalasia patients (table 2).

In RS+ achalasia patients, acidification was more often due to acid fermentation compared with those without reflux symptoms (RS+: mean 6.6%, 95% CI 3.0% to 10.2% versus RS−: 1.8%, 95% CI −0.45% to 4.1%; p=0.03) and acid reflux with delayed clearance was also more often seen in these patients (RS+: mean 6%, 95% CI 0.94% to 11.0% versus RS−: 3.4%, 95% CI −0.34% to 7.2%, p=0.051; table 2). In RS− achalasia patients, the dominant acidification pattern was stasis of ingested acidic food or drink (RS+: mean 2.3%, 95% CI −0.04% to 4.6% v versus RS−: 7.6%, 95% CI −0.12% to 15.3%; p=0.18; table 2).

During 24-hour pH-impedance monitoring the total number of reported reflux symptoms for all patients in the RS+ group was 84, compared with 7 symptom episodes in the RS− group (RS+: median symptoms per patient 4 (3–5) vs RS−: 0 (0–2), p<0.001). Not a single patient had a SI of ≥50%, indicating poor specificity of their symptoms for acidification events. In the RS+ group, symptoms with a positive association were related to acid reflux with delayed clearance. All symptoms in the RS− group had a negative symptom correlation.

No difference in baseline impedance levels was observed between the RS+ and RS− groups (table 2).

### Postprandial stationary HRM and pH-impedance measurement

The postprandial HRM and pH-impedance measurement revealed no difference in acid exposure or acidification patterns between RS+ and RS− achalasia patients (table 3). Only 18% (n=7/38) of all patients had acid exposure at all during the 2-hour postprandial measurement. In these patients (RS+: n=5 vs RS−: n=2), acid exposure was due to acid reflux with normal or delayed clearance. Prolonged acidification was only seen in two patients, one patient in each group. None of the reflux episodes were detected by the proximal pH probe. Low baseline impedance tracings prevented exact localisation of the proximal extent of each reflux episode, based on the position of the proximal pH probe is was at least below 10 cm. The main mechanism associated with these reflux episodes was swallow-induced reflux in both groups. Transient LOS relaxations were not observed at all. In both groups, none of the reported symptoms during the 2 hour postprandial measurement were related to reflux or acid exposure.

**Table 3:** Outcome of postprandial combined HRM and pH-impedance monitoring of treated achalasia patients with (RS+) and without reflux symptoms (RS−)

	Patients with reflux symptoms (RS+)	Patients without reflux symptoms (RS−)	P value
	(n=19)	(n=19)	
Postprandial combined HRM and pH-impedance measurement			
Presence acid exposure (n (%))	5/19 (26)	2/19 (11)	0.41
Acid exposure time (AET: % of time pH <4; mean (95% CI)	1.9 (−1.06 to 4.85)	2.6 (−2.67 to 7.87)	0.81
Acidification patterns (% of time; mean (95% CI))			
Acid reflux with normal clearance	0.3 (−0.06 to 0.78)	0 (0 to 0)	0.09
Acid reflux with delayed clearance	1.4 (−1.48 to 4.18)	2.5 (−2.78 to 7.80)	0.69
Acid fermentation	0 (0 to 0)	0 (0 to 0)	1
Stasis of ingested acidic food	0 (0 to 0)	0 (0 to 0)	1
Unclassified	0.2 (−0.21 to 0.60)	0 (0 to 0)	0.53
No of acidification events (median (IQR))			
Acid reflux with normal clearance	0 (0–2)	0 (0–0)	0.04
Acid reflux with delayed clearance	0 (0–1)	0 (0–1)	0.97
Acid fermentation	0 (0–0)	0 (0–0)	1
Stasis of ingested acidic food	0 (0–0)	0 (0–0)	1
Unclassified	0 (0–1)	0 (0–1)	0.32
No of patients per acidification pattern (n (%))			
Acid reflux with normal clearance	4 (21)	0 (0)	0.04
Acid reflux with delayed clearance	1 (5)	1 (5)	1
Acid fermentation	0 (0)	0 (0)	1
Stasis of ingested acidic food	0 (0)	0 (0)	1
Unclassified	1 (5)	0 (0)	0.32
No of symptoms (median (IQR))	0 (0–1)	0 (0–0)	0.02

AET, acid exposure time; HRM, high-resolution manometry.

### Oesophagogastroduodenoscopy

The presence of reflux oesophagitis during oesophagogastroduodenoscopy was not significantly different between RS+ and RS– achalasia patients (table 4). In both groups, the severity of reflux oesophagitis, when present, was classified as grade A or B.

**Table 4:** Results of oesophageal function tests, endoscopy and questionnaires in treated achalasia patients with (RS+) and without reflux symptoms (RS–)

	Patients with reflux symptoms (RS+)	Patients without reflux symptoms (RS–-)	P value
	(n=19)	(n=19)	
High-resolution manometry			
Basal LOS pressure (mm Hg, median (IQR))	3 (2–6)	3 (3–6)	0.88
Integrated relaxation pressure (mm Hg, median (IQR))	6.2 (2.1–8.7)	5.9 (4.1–9)	0.87
Classification of oesophageal contractility			1
Failed contractility (n (%))	13 (68)	13 (68)	
Weak contractility (n (%))	6 (32)	6 (32)	
OGJ distensibility (at 50 mL: mm^2^/mm Hg, median (IQR))	5.3 (4.5–6.9)	5.3 (4.5–6.9)	0.18
Timed barium oesophagogram			
Barium column at 5 min (cm, median (IQR))	1 (0–2)	1.8 (0–2.5)	0.34
Barium column at 2 min	1.6 (0–2)	2.4 (1–3.5)	0.1
Oesophageal diameter (cm, median (IQR))	2.1 (1.8–3)	2.5 (2–3.4)	0.12
Endoscopy			
Reflux oesophagitis (n (%))	10 (53)	4 (21)	0.91
Grade A	5/10 (50)	2/4 (50)	
Grade B	5/10 (50)	2/4 (50)	
Questionnaires (median (IQR))			
RDQ total score*	1.9 (1.4–3.3)	0.3 (0–0.6)	<0.001
Heartburn	3 (2–4.3)	0 (0–1)	<0.001
Regurgitation	1 (0.8–2.8)	0 (0–0.3)	<0.001
Dyspepsia	1.8 (0–4)	0 (0–0.5)	<0.002
GORD	2.1 (1.5–2.8)	0.3 (0–0.8)	<0.001
Achalasia-DSQoL†	19 (14–22)	13 (12–16)	<0.005
SF-36‡			
Physical component summary score	51 (46–55)	54 (51–58)	0.06
Mental component summary score	57 (53–60)	54 (53–58)	0.37

*RDQ: 12-item questionnaire, providing a score for each typical reflux symptom on a Likert scale, range 0 to5. Per domain the mean score was calculated per patient.

†Achalasia-DSQoL: quality of life related to achalasia, range 10 to 33, lower score indicated better quality of life.

‡SF-36 score consisted of a physical and mental component summary score, each ranged from 0 to 100, with higher scores indicating better quality of life.

DSQoL, Disease-Specific Quality-of-Life questionnaire; GORD, gastro-oesophageal reflux dimension; LOS, lower oesophageal sphincter; OGJ, oesophagogastric junction; RDQ, Reflux Disease Questionnaire; SF-36, 36-item Short-Form Health Survey.

### Provocation tests: acid perfusion and oesophageal distension

**Figure 3:**
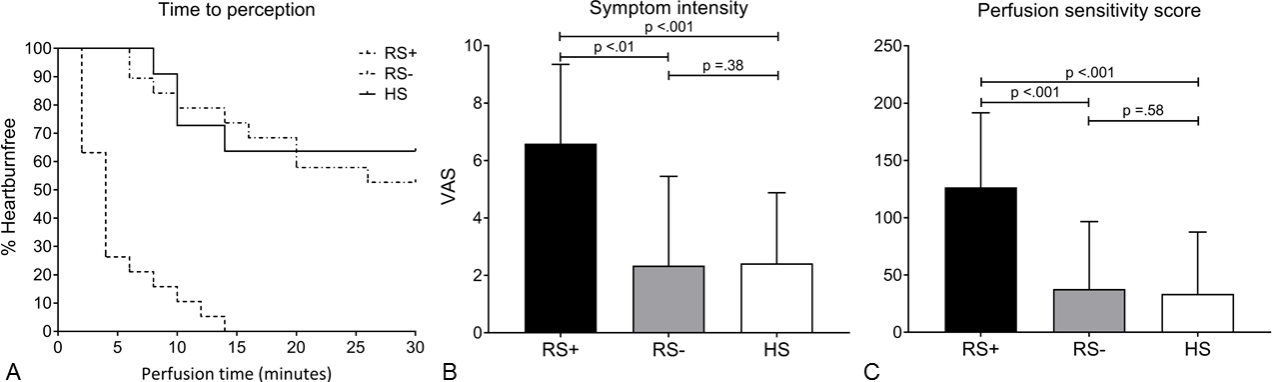
Results of acid perfusion test in treated achalasia patients with reflux symptoms (RS+), without reflux symptoms (RS–) and healthy subjects (HS). (A) Lag time to initial heartburn perception. (B) Mmaximum symptom intensity expressed by Visual Analogue Scale (VAS). (C) Perfusion sensitivity score ((total perfusion time—lag time to perception) X VAS).

The outcome parameters of the acid perfusion test are shown in figure 3. RS+ achalasia patients were much more sensitive to acid perfusion, as evidenced by a shorter time to perception of heartburn compared with the RS– achalasia patients and normal values of healthy subjects (RS+: median 4 (2–6) min; RS–: 30 (14–30) min; HS: 30 (10–30) min, log rank p<0.001). Sensitivity values of RS– achalasia patients were comparable to healthy subjects. The perceived symptom intensity for heartburn or discomfort was also significantly higher in the RS+ group compared with the RS– group and healthy subjects (RS+: median VAS 7 (4.8–9) vs RS–: VAS 0.5 (0–4.5); RS+: VAS 7 (4.8–9) vs HS: VAS 1.6 (0.4–2.4), both p<0.001). Consequently, the perfusion sensitivity score was significantly higher in the RS+ group compared with the RS– group and healthy subjects (RS+: median 139 (65–173) vs RS–: 0 (0–99); RS+: 139 (65-173) vs HS: 0 (0–92), both p<0.001). The scores for symptom intensity and perfusion sensitivity of RS– achalasia patients were similar to the scores of healthy subjects. In 26% (5/19) of the RS+ achalasia patients and 11% (2/19) of the RS– achalasia patients, the acid perfusion test was prematurely stopped due to intolerable pain.

In one patient, oesophageal distension by EndoFLIP could not be performed due to vasovagal syncope during the measurement. In 22% (4/18) of the RS+ and in 5% (1/19) of the RS– achalasia patients, pain and discomfort prevented full completion of the distension protocol. Perception of distension in RS+ achalasia patients occurred at a lower balloon volume (RS+: median 50 (38–70) mL vs RS–: 70 (50–70) mL, p=0.03) and with a higher intensity (RS+: median VAS 3 (1.1–7.4) vs RS–: VAS 0 (0–2.8), p=0.03). The distension score was significantly higher in RS+ achalasia patients (RS+: median 47 (0–166) vs RS–: 0 (0–18), p=0.03).

All RS+ achalasia patients had a decreased perception threshold to acid and 67% (12/18) had a decreased perception threshold for mechanical distension, indicating visceral hypersensitivity in these patients. In contrast, 53% (10/19) of RS– achalasia patients did not experience any heartburn or discomfort during acid perfusion and 68% (13/19) lacked any symptoms during distension.

### HRM, timed barium oesophagogram and OGJ distensibility

All patients completed the HRM, timed barium oesophagogram and OGJ distensibility measurements, data are shown in table 4. No differences were observed in OGJ distensibility and outcome parameters of HRM and timed barium oesophagogram between achalasia patients with and without reflux symptoms.

### Questionnaires

Outcomes of the questionnaires are presented in tables 1 and 4. The GERDQ and RDQ scores were significantly higher in RS+ achalasia patients. The Eckardt score and general quality of life, scored by the SF-36, were similar between the groups. Achalasia-related quality of life was significantly decreased in RS+ patients, reflected by a higher overall score.

### Factors related to acid hypersensitivity, acid exposure and acidification patterns

When lumping all patients regardless of presence of reflux symptoms, patients with hypersensitivity to acid perfusion (n=25/38, 66%) did not have higher acid exposure times or more often reflux oesophagitis compared with achalasia patients without hypersensitivity to acid perfusion (online supplementary 1, table 1). Outcomes of baseline impedance, oesophageal function and emptying were also not significantly different (online supplementary 1, table 1). However, patients with acid hypersensitivity were also more sensitive to mechanical distension, which is reflected by a perceived perception at a lower balloon volume (median balloon volume 50 (40–70) mL vs 70 (65–70) mL, p=0.01) and a higher distension sensitivity score (median 47 (0–160) vs 0 (0–7), p=0.02).

Statistically significant correlations were found between acid exposure and OGJ distensibility (r=0.403, p=0.012), basal LOS pressure (r=−0.348, p=0.032) and barium column height during timed barium oesophagogram (r=−0.347, p=0.033). All patients with pathological acid exposure, independent of reflux symptoms, had an increased OGJ distensibility, lower basal LOS pressure and an adequate oesophageal emptying (online supplementary 1, table 2).

Analysing the acidification patterns in absence of reflux symptoms revealed significant negative correlations between barium column height at 5 min during timed barium oesophagogram and either acid reflux with delayed clearance (OR 0.53 (95% CI 0.3 to 0.93), p=0.03) or acid fermentation (OR 0.52, 95% CI 0.29 to 0.93, p=0.03). Stasis of ingested acidic food or drink showed the opposite, a positive correlation with barium column height was observed, but this correlation was not significant (OR 1.4, 95% CI 0.95 to 2.1, p=0.09). Other outcome parameters (eg, OGJ distensibility, IRP and Eckardt score) were not significantly correlated with the different acidification patterns.

### Subgroup analysis of outcome parameters per treatment group

Online supplementary 1, table 3 shows the outcome of the 24-hour impedance measurement, oesophagogastroduodenoscopy and provocation tests according to treatment, laparoscopic Heller’s myotomy versus POEM. Total and supine acid exposure were significantly increased in achalasia patient treated by POEM compared with laparoscopic Heller’s myotomy. However, the number of patients with pathological acid exposure was comparable. No differences were observed in acidification patterns, reflux oesophagitis or chemical and mechanical oesophageal sensitivity. The observed acidification was in both treatment groups mainly determined by acid reflux with delayed clearance, acid fermentation and stasis of ingested acidic food.

## Discussion

This study was designed to allow a thorough investigation of reflux symptoms in treated achalasia patients and to increase the understanding of the underlying mechanisms by analysing oesophageal function, acid exposure, acidification patterns, symptom perception and reflux oesophagitis. The most important findings made in this study are that reflux symptoms in treated achalasia patients are rarely caused by gastro-oesophageal reflux and that oesophageal hypersensitivity to chemical (acid) and mechanical (distension) stimuli is likely to play a substantial role.

Treatment of achalasia focuses on disrupting the LOS, compromising the barrier against reflux of gastric content. The reported prevalence of presumed reflux-related complications after achalasia treatment is variable, ranging from 5% to 60%.^2–6^ In part, this variability is likely to be related to treatment type, with lower occurrence rates after pneumodilation (5%–25%) and higher rates after laparoscopic or endoscopic myotomy (20%–60%).^2–9^ However, the reported prevalence of gastro-oesophageal reflux after achalasia treatment also depends on the criteria used to define ‘reflux’. Most studies used presence of reflux symptoms and/or presence of oesophagitis, whereas it has been shown that, in treated achalasia, there is considerable discordance between reflux symptoms, oesophageal acid exposure as measured with pH monitoring and presence of oesophagitis.^2 6 10–15^ It has also been put forward that combined pH-impedance monitoring, as was used in our study, is essential to differentiate between true reflux, stasis and fermentation.^10 12^ Overestimation of the role of gastro-oesophageal reflux in these patients led to prescribing PPI as the standard treatment, which has a variable efficacy as it treats acid reflux but not acidification of oesophageal contents by other causes. The advent of POEM, which has been shown to be associated with a high postprocedural prevalence of oesophagitis, further underlines the need of better understanding of this problem.^3 4 8^ The present study had the objective to provide a complete image of reflux-related factors involved in the generation of post-treatment reflux symptoms and signs in achalasia patients by analysing oesophageal function, acid exposure, acidification patterns, symptom perception and mucosal status. To our knowledge, equally extensive studies on this subject have not been performed thus far.

Our study has confirmed that pathological acid exposure, defined as time with oesophageal pH <4 greater than 6%, is very common in treated achalasia patients (63% (n=24/38) of patients) with a comparable frequency after laparoscopic Heller’s myotomy with Dor fundoplication (55% (n=11/20)) as POEM (81% (n=13/16)). However, the results of our study also show that this is not predominantly caused by acid reflux with normal or delayed clearance but that it is largely due to other mechanisms, such as acid fermentation and stasis of ingested acidic food, resulting in oesophageal acidification. Furthermore, the prevalence of pathological acid exposure was not significantly different in treated achalasia patients with and without reflux symptoms (74% and 53%, respectively). Most importantly, however, in none of the 19 patients with reflux symptoms, a positive temporal association between acidification events and symptom episodes could be demonstrated. Since the results of this study strongly support the notion that reflux symptoms in treated achalasia patients are not primarily related to (increased) gastro-oesophageal reflux, treatment with a PPI is likely to be ineffective in most of these patients.

Treated achalasia patients with reflux symptoms had a higher sensitivity to acid perfusion and to mechanical distension than patients without reflux symptoms. Patient characteristics, such as achalasia subtype, type of treatment and disease duration, seemed not to influence enhanced sensitivity. Thus far, evaluation of acid sensitivity in achalasia patients with reflux symptoms has only been performed in untreated patients and showed that the prevalence of oesophageal acid sensitivity was lower in these patients compared with a group of patients with GORD.^33^ This could suggest that the content of acid and its volume influences oesophageal sensitivity, which is previously described.^34 35^ However, acid hypersensitivity is also present in patients with non-erosive reflux disease, a group of patients with fewer reflux episodes and acid exposure compared to patients with GORD.^34 36^ In these patients, acid hypersensitivity seems associated with impaired mucosal integrity, increased activation of oesophageal nociceptors and visceral sensitisation, peripherally or centrally mediated.^19 37 38^ All of the five acidification patterns described in this paper—acid reflux with normal clearance, acid reflux with delayed clearance, acid fermentation, prolonged oesophageal acidity after ingestion of acidic food and unclassified acidity—might act as triggers for the development of peripheral and central sensitisation. Hypothetically, sensitisation in achalasia patients, treated or untreated, could also be evoked by stasis of non-acid food remnants. Furthermore, it has been demonstrated that a difference in psychological perception of anxiety and stress can also influence visceral sensitivity.^39 40^ The relation between psychological stressors and chemical or mechanical oesophageal perception was not analysed in this study. Given the conceptual importance of hypersensitivity in treated achalasia patients with reflux-like symptoms, studies exploring the efficacy of visceral analgetics such as citalopram or amitriptyline seem warranted.

In contrast to the observed chemical and mechanical hypersensitivity, previous studies describe hyposensitivity to these stimuli in achalasia patients post-treatment.^39 41^ The pathophysiology of the described hyposensitivity in achalasia is incompletely understood. It is hypothesised that in addition to motor neuron loss, sensory neurons are affected and/or desensitised, especially in longstanding disease.^39^ Although, achalasia patients without reflux symptoms demonstrated decreased chemical and mechanical sensitivity compared with the symptomatic patients, no difference in the outcome of the acid perfusion test with healthy subjects was observed. In addition, no difference in disease duration, achalasia subtype or treatment was seen. Based on these data, it cannot indisputable be concluded that oesophageal hyposensitivity explains the absence of symptoms in the asymptomatic reflux group.

Among the four patterns leading to prolonged acidification in achalasia patients, acid fermentation of oesophageal food residues has gained most attention in previous studies.^10 12 42^ In their in vitro study, Crookes *et al* observed that the pH of saliva incubated with chewed food at body temperature slowly drifted to a median pH of 4, in a period of approximately 6 hours.^10^ The acid fermentation observed in our study showed a more rapid pH drift and often reached values below 4, with the lowest pH ranging from 3 to 1. We propose that the quicker pH drop observed in our study may be the result of, the contribution of bacterial overgrowth in the oesophagus leading to a quicker fermentation process and prolonged delayed clearance in supine position. In addition, it cannot completely be excluded that some pH drops, interpreted as acid fermentation, are the result of pH drift, or contact of the pH electrode with small particles of acidic food or stomach content. However, we feel that the use of an ISFET pH electrode makes pH drift as a cause of the phenomenon unlikely. Of the other three acidification patterns, acidic food-induced stasis could be implicative of failed treatment and diagnostics to evaluate oesophageal clearance should be considered.

Baseline impedance levels were substantially reduced in all achalasia patients, which made us decide not to use impedance for the classification of acidification patterns. No correlations were observed between baseline impedance levels and acid exposure or acid hypersensitivity. Low baseline impedance levels are common in achalasia patients and caused by stasis of luminal content, dilated oesophageal lumen and ineffective motility leading to ineffective clearance and mucosal damage.^43 44^ Although interpretation of impedance can be difficult in achalasia patient it helped to identify prolonged acidification, clearance of acidification and air trapping. The use of pH-impedance monitoring is therefore essential for understanding acidification in achalasia patients.

This study shows that the causes underlying reflux symptoms in treated achalasia are diverse. For an adequate diagnosis and tailored treatment of these symptoms, a stepwise approach is advised that starts with an oesophagogastroduodenoscopy. When reflux oesophagitis is observed, acid suppression should be started combined with lifestyle advice. In case of persistent symptoms or absent reflux oesophagitis, a 24-hour pH-impedance monitoring should be performed to assess the relative contribution of the various mechanisms leading to oesophageal acidification. Acid reflux with normal and delayed clearance can be treated by increasing the PPI dose or adding an H2-recept antagonist. When acid fermentation predominates, avoidance of meals shortly before bedtime and drinking water after meals may be advised. In case symptoms persist, acid hypersensitivity should be considered in both groups and a perception-modulating antidepressant could be considered. Patients with pathological acid exposure due to acidic food-induced stasis or physiological or absent acid exposure should undergo a timed barium oesophagogram to evaluate oesophageal emptying or an OGJ distensibility measurement. If oesophageal clearance or OGJ distensibility is severely impaired, retreatment for achalasia may be considered. For patients with physiological or absent acid exposure and adequate oesophageal clearance, a therapeutic trial that aims to reduce oesophageal hypersensitivity can be considered.

In conclusion, reflux symptoms in treated achalasia patients are rarely caused by gastro-oesophageal reflux and most instances of oesophageal acidification in these patients are not reflux induced. Rather, increased oesophageal sensitivity to chemical and mechanical stimuli may determine the generation of reflux symptoms in these subjects. These observations have implications for the management of treated achalasia patients with reflux-like symptoms.

10.1136/gutjnl-2020-320772.supp2 Supplementary data
