# Supplementary material for: Reflux symptoms and oesophageal acidification in treated achalasia patients are often not reflux related
Source: Gut. 2020 May 21;70(1):30–9. doi: 10.1136/gutjnl-2020-320772 (PMC7788183; doi:10.1136/gutjnl-2020-320772)
Supplement: Supplementary data [file gutjnl-2020-320772supp001.pdf]

**Supplement 1**

This appendix has been provided by the authors to give readers additional information about the study.

# Reflux symptoms and oesophageal acidification in treated achalasia patients are often not reflux related

Fraukje A. Ponds, Jac M. Oors, André J.P.M. Smout, Albert J. Bredenoord

*Department of Gastroenterology and Hepatology, Amsterdam UMC, University of Amsterdam, Meibergdreef 9, Amsterdam, The Netherlands.*



Ponds FA, *et al*. *Gut* 2020; 70:30–39. doi: 10.1136/gutjnl-2020-320772

**Appendix**

1. Study subject and inclusion criteria

    1.1 Eligibility criteria

**Tables**

1. Factors related to acid hypersensitivity.
2. Factors related to pathological acid exposure.
3. Outcome 24-hour pH-impedance, oesophagogastroduodenoscopy and provocation tests per treatment group.

## 1. Study subject and inclusion criteria

### *1.1 Eligibility criteria*

**Inclusion criteria**

Treated achalasia patients with gastro-oesophageal reflux symptoms

- Diagnosis of idiopathic achalasia confirmed by oesophageal manometry that shows the following criteria:
    - Aperistalsis or simultaneous contractions in the oesophageal body.
    - LOS dysrelaxation.
- Treatment of achalasia with one of the following procedures:
    - Endoscopic balloon dilatation
    - Surgical Heller myotomy
    - Per-oral endoscopic myotomy (POEM)
- Minimum total score on the Gastro-Oesophageal Reflux Disease Questionnaire (GORDQ) of ≥ 8.
- Gastro-oesophageal symptoms after treatment lasting more than 3 months.
- Age 18-80 years.
- Written informed consent.

Treated achalasia patients without gastro-oesophageal reflux symptoms

- Diagnosis of idiopathic achalasia confirmed by oesophageal manometry that shows the following criteria:
    - Aperistalsis or simultaneous contractions in the oesophageal body.
    - LOS dysrelaxation.
- Treatment of achalasia with one of the following procedures:
    - Endoscopic balloon dilatation
    - Surgical Heller myotomy
    - Per-oral endoscopic myotomy (POEM)
- Maximum total score on the Gastro-Oesophageal Reflux Disease Questionnaire (GORDQ) of < 8.
- No gastro-oesophageal symptoms after treatment.
- Age 18-80 years.



Ponds FA, *et al. Gut* 2020; 70:30–39. doi: 10.1136/gutjnl-2020-320772

Supplemental material BMJ Publishing Group Limited (BMJ) disclaims all liability and responsibility arising from any reliance placed on this supplemental material which has been supplied by the author(s) Gut



- Written informed consent.

**Exclusion criteria**

Treated achalasia patients with gastro-oesophageal reflux symptoms

- Pseudoachalasia.
- Upper gastrointestinal malignancy.
- Chagas disease.
- Peptic ulcer disease.
- Inability to stop PPI, H2-receptor antagonist or prokinetic drug for two weeks
- Presence of an extremely dilated oesophagus body >5 cm

Treated achalasia patients without gastro-oesophageal reflux symptoms

- Pseudoachalasia.
- Upper gastrointestinal malignancy.
- Chagas disease.
- Peptic ulcer disease.
- Inability to stop PPI, H2-receptor antagonist or prokinetic drug for two weeks
- Presence of an extremely dilated oesophagus body >5 cm



Ponds FA, *et al. Gut* 2020; 70:30–39. doi: 10.1136/gutjnl-2020-320772

**Tables**

| | Patients with acid hypersensitivity (n=25) | Patients without acid hypersensitivity (n=13) | *P* |
|---|---|---|---|
| **Achalasia subtype at diagnosis** (n (%)) | | | 0.64 |
| Type I | 9 (36) | 4 (31) | |
| Type II | 15 (60) | 8 (62) | |
| Type III | 1 (4) | 1 (8) | |
| **Achalasia treatment** (n (%)) | | | 0.90 |
| Pneumodilation# | 1 (4) | 1 (8) | |
| Laparoscopic Heller's myotomy | 7 (28) | 2 (15) | |
| Peroral endoscopic myotomy | 7 (28) | 4 (31) | |
| Pneumodilation* and laparoscopic Heller's myotomy | 7 (28) | 4 (31) | |
| Pneumodilation* and peroral endoscopic myotomy | 3 (12) | 2 (15) | |
| **Disease duration** (years (mean (SD)) | 8.2 (7.0) | 7.8 (5.7) | 0.85 |
| **24-hour pH-impedance monitoring** | | | |
| **Acid exposure time** (AET: % of time pH <4; mean (95% CI)) | | | |
| Total | 13.9 (7.3 to 20.5) | 9.2 (3.4 to 14.9) | 0.33 |
| Upright | 10.8 (5.3 to 16.2) | 4.8 (2.2 to 7.4) | 0.05 |
| Supine | 18.0 (7.7 to 28.4) | 15.2 (4.1 to 26.3) | 0.73 |
| **Pathological acid exposure** (AET pH<4 in >6%; n (%)) | 17 (68) | 7 (54) | 0.39 |
| **Baseline impedance** (Ω; median (IQR)) | | | |
| Proximal | 2411 (1649-3150) | 2220 (1780-2773) | 0.55 |
| Distal | 487 (368-660) | 476 (339-750) | 1.00 |
| **Endoscopy** | | | |
| Reflux oesophagitis (n (%) | 11 (44) | 3 (23) | 0.29 |
| **High resolution manometry** | | | |
| Basal LOS pressure (mmHg, median (IQR)) | 3 (2-6) | 3 (3-7) | 0.70 |
| Integrated relaxation pressure (mmHg, median (IQR)) | 6.6 (3.3-8.6) | 5.9 (3.7-9.3) | 0.87 |
| **OGJ distensibility** (at 50 mL, mmHg/m$^2$, median (IQR)) | 5.2 (4.5-7.0) | 4.8 (2.7-5.8) | 0.11 |

| | | | |
|---|---|---|---|
| **Timed barium oesophagogram** | | | |
| Barium column at 5 min (cm, median (IQR)) | 1.4 (0-2.5) | 1.7 (0-2.6) | 0.81 |
| Oesophageal diameter (cm, median (IQR)) | 2.5 (2-3.1) | 2.3 (2-2.8) | 0.93 |
| **Perception oesophageal mechanical distension** | | | |
| Volume first perception (mL, median (IQR)) | 50 (40-70) | 70 (65-70) | 0.01 |
| Symptom intensity (VAS, median (IQR)) | 2.9 (0.3-5.9) | 0 (0-1.9) | 0.05 |
| Distension sensitivity score (median (IQR)) | 47 (0-160) | 0 (0-7) | 0.02 |

**Table 1.** Factors related to acid hypersensitivity.

Abbreviations: AET, acid exposure time; CI, confidence interval; LOS, lower oesophageal sphincter; OGJ, oesophagogastric junction; IQR, interquartile range; VAS, visual analogue score.
#Pneumodilation up till 35-mm balloon. *Pneumodilation up till 40-mm balloon.

| | Patients with pathological acid exposure (n=24) | Patients without pathological acid exposure (n=14) | *P* |
|---|---|---|---|
| **BMI** (kg/m$^2$; mean (SD)) | 26.2 (4.3) | 24.8 (2.7) | 0.27 |
| **High resolution manometry** | | | |
| Basal LOS pressure (mmHg, median (IQR)) | 3 (2-4.8) | 4.6 (3-9.3) | 0.034 |
| Integrated relaxation pressure (mmHg, median (IQR)) | 6.2 (3.1-8.7) | 6.4 (4-10.5) | 0.56 |
| **OGJ distensibility** (at 50 mL, mmHg/m$^2$, median (IQR)) | 5.5 (4.6-7.0) | 4.6 (3.4-5.5) | 0.032 |
| **Timed barium oesophagogram** | | | |
| Barium column at 5 min (cm, median (IQR)) | 0 (0-1.8) | 2.5 (1.2-3.1) | 0.002 |
| Oesophageal diameter (cm, median (IQR)) | 2.2 (2-3) | 2.7 (2-3.4) | 0.18 |
| **24-hour pH-impedance monitoring** | | | |
| **Baseline impedance** (Ω, median (IQR)) | | | |
| Proximal | 2163 (1865-2739) | 2763 (1585-3260) | 0.23 |
| Distal | 487 (347-730) | 493 (348-702) | 0.88 |
| **Acid perfusion test** | | | |
| Time to perception (min, median (IQR)) | 8 (4-27.5) | 9 (5.5-30) | 0.46 |
| Symptom intensity (VAS, median (IQR)) | 5.5 (0.6-8) | 4.1 (0-7) | 0.48 |
| Perfusion hypersensitivity score (median (IQR)) | 82 (1-135) | 64 (0-170) | 0.82 |
| **Perception oesophageal mechanical distension** | | | |
| Volume first perception (mL, median (IQR) | 70 (50-70) | 60 (40-70) | 0.60 |
| Symptom intensity (VAS, median (IQR)) | 1.5 (0-4.3) | 1 (0-5.2) | 0.82 |
| Oesophageal distension sensitivity score (median (IQR)) | 0 (0-84) | 9 (0-174) | 0.61 |

**Table 2.** Factors related to pathological acid exposure.

Abbreviations: BMI, body mass index; CI, confidence interval; LOS, lower oesophageal sphincter; IQR, interquartile range; OGJ, Oesophagogastric junction; VAS, visual analogue score.



Ponds FA, *et al. Gut* 2020; 70:30–39. doi: 10.1136/gutjnl-2020-320772

| | Laparoscopic Heller's myotomy (n=20) | Peroral endoscopic myotomy (n=16) | *P* |
|---|---|---|---|
| **24-hour pH-impedance monitoring** | | | |
| **Acid exposure time** (AET: % of time pH <4; mean (95% CI)) | | | |
| Total | 8.1 (3.6 to 12.5) | 19.1 (10.2 to 28.1) | 0.02 |
| Upright | 6.5 (2.3 to 10.7) | 12.5 (5.4 to 19.6) | 0.12 |
| Supine | 9.6 (3.4 to 15.9) | 28.5 (13.3 to 43.7) | 0.02 |
| **Pathological acid exposure** (AET pH<4 in >6%; n (%)) | 11 (55) | 13 (81) | 0.16 |
| **Acidification patterns** (% of time; mean (95% CI)) | | | |
| Acid reflux with normal clearance | 0.08 (0.01 to 0.14) | 0.2 (0.06 to 0.33) | 0.10 |
| Acid reflux with delayed clearance | 2.8 (0.07 to 5.57) | 7.6 (1.1 to 14.1) | 0.16 |
| Acid fermentation | 3.5 (0.8 to 6.12) | 5.6 (1.4 to 9.8) | 0.34 |
| Stasis of ingested acidic food | 3.8 (-1.04 to 8.58) | 6.7 (-0.9 to 14.6) | 0.46 |
| Unclassified | 0.3 (-0.1 to 0.73) | 1.8 (-0.98 to 4.6) | 0.28 |
| **Number of acidification events** (median (IQR)) | | | |
| Acid reflux with normal clearance | 0 (0-1) | 0.5 (0-2.8) | 0.15 |
| Acid reflux with delayed clearance | 0 (0-1) | 1.5 (0-3) | 0.07 |
| Acid fermentation | 0 (0-1.8) | 0.5 (0-2.5) | 0.57 |
| Stasis of ingested acidic food | 0 (0-1) | 0 (0-2) | 0.23 |
| Unclassified | 0 (0-1) | 0 (0-0.8) | 0.79 |
| **Number of patients per acidification pattern** (n (%)) | | | |
| Acid reflux with normal clearance | 6 (30) | 8 (50) | 0.22 |
| Acid reflux with delayed clearance | 8 (40) | 10 (63) | 0.18 |
| Acid fermentation | 8 (40) | 9 (56) | 0.33 |
| Stasis of ingested acidic food | 6 (30) | 7 (44) | 0.39 |
| Unclassified | 6 (30) | 4 (25) | 0.74 |
| **Endoscopy** | | | |
| Reflux oesophagitis (n (%)) | 7 (35) | 7 (44) | 0.59 |
| **Acid perfusion test** | | | |
| Time to perception (min, median (IQR)) | 6 (4-20) | 14 (4-30) | 0.26 |
| Symptom intensity (VAS, median (IQR)) | 5.4 (1.1-7.8) | 3.8 (0-7.9) | 0.65 |
| Perfusion sensitivity score (median (IQR)) | 112 (8-172) | 35 (0-110) | 0.12 |

| | | | |
|---|---|---|---|
| **Perception oesophageal mechanical distension** | | | |
| Volume first percepton (mL, median (IQR) | 60 (40-70) | 70 (50-70) | 0.36 |
| Symptom intensity (VAS, median (IQR)) | 3 (0-5.2) | 1.2 (0-2.6) | 0.26 |
| Distension sensitivity score (median (IQR)) | 13 (0-170) | 0 (0-71) | 0.26 |

Table 3. Outcome 24-hour pH-impedance, oesophagogastroduodenoscopy and provocation tests per treatment group.

Abbreviations: AET, acid exposure time, CI, confidence interval; IQR, interquartile range; VAS, visual analogue score.
