# Supplementary material for: Reflux symptoms and oesophageal acidification in treated achalasia patients are often not reflux related
Source: Gut. 2020 May 21;70(1):30–9. doi: 10.1136/gutjnl-2020-320772 (PMC7788183; doi:10.1136/gutjnl-2020-320772)
Supplement: Supplementary data [file gutjnl-2020-320772supp002.pdf]

Research protocol

Reflux symptoms in patients with achalasia

Correspondence to:
Drs. F.A.M. Ponds
Motiliteitscentrum, MDL
Academic Medical Centre, C2-231
Meidreef 5
1105 AZ Amsterdam

Version number: 02
Date: 17 January 2013

Ponds FA, *et al. Gut* 2020; 70:30–39. doi: 10.1136/gutjnl-2020-320772

PROTOCOL TITLE

'Reflux symptoms in patients with achalasia'

**Protocol ID**	**NL42367.018.12**
**Short title**	**Reflux symptoms in achalasia**
**Version**	**1**
**Date**	**12-10-2012**
**Coordinating investigator(s) / project leader(s)**	**1. Dr. A.J. Bredenoord<br>C2-325<br>a.j.bredenoord@amc.uva.nl<br>tel: 020 566 1745<br>2. Drs. F.A.M. Ponds<br>C2-231<br>f.a.ponds@amc.uva.nl<br>tel: 020 566 464**
**Principal investigator(s) (in Dutch: hoofdonderzoeker/uitvoerder)**	**Prof. Dr. A.J.P.M. Smout<br>C2-326<br>a.j.smout@amc.uva.nl<br>tel: 020 566 8708**
**Sponsor (in Dutch: verrichter/opdrachtgever)**	**Afdeling MDL<br>AMC Amsterdam, C2<br>Meibergdreef 9<br>1105 AZ Amsterdam**
**Independent physician(s)**	**Dr. M. Lowenberg<br>C2-325<br>m.lowenberg@amc.uva.nl<br>tel: 020 566 7621**
**Laboratory sites**	**AMC Amsterdam**

Ponds FA, *et al. Gut* 2020; 70:30–39. doi: 10.1136/gutjnl-2020-320772

PROTOCOL SIGNATURE SHEET

Name	Signature	Date
**Head of Department:**<br><br>**Prof. Dr. P. Fockens**		
**Principal Investigator:**<br><br>**Prof. Dr. A.J.P.M. Smout**		

TABLE OF CONTENTS

1. INTRODUCTION AND RATIONALE 6
2. OBJECTIVES 7
3. STUDY DESIGN 8
4. STUDY POPULATION 8
4.1 Population (base) and study enrolment 8
4.2 Inclusion criteria 9
4.3 Exclusion criteria 9
4.4 Sample size calculation 10
5. METHODS 10
5.1 Study parameters/endpoints 10
5.1.1 Main study parameter/endpoint 10
5.1.2 Secondary study parameters/endpoints 10
5.2 Study procedures 11
5.2.1 Research protcol 11
5.2.2 Procedures and questionnaire 13
5.3 Withdrawal of individual subjects 15
6. SAFETY 16
6.1 Section 10 WMO event 16
6.2 Adverse events and serious adverse events 16
6.3 Follow-up adverse events 16
6.4 Annual safety report 16
7. PRIVACY 17
8. STASTICAL ANALYSIS 17
9. ETHICAL CONSIDERATIONS 17
9.1 Regulation statement 17
9.2 Recruitment and consent 17
9.3 Benefits and risks assessment 18
9.4 Compensation for injury 18
9.5 Incentives 18
10. ADMINISTRATIVE ASPECTS AND PUBLICATION 18
10.1 Handeling and storage of data and documents: biobankregulations 18
10.2 Amendments 19
10.3 End of study report 19
11. REFERENCES 19

SUMMARY

Rationale: Achalasia is a rare motility disorder of the esophagus that is characterised by aperistalsis of the esophageal body and dysrelaxation of the lower esophageal sphincter (LES). Current treatment is palliative and the aim of the treatment is to diminish the obstructive function of the esophagogastric junction (EGJ). Due to this approach the most frequent complication post-treatment is gastroesophageal reflux (GER). However, not every treated patient develops GER symptoms and the mechanism behind the occurrence of GER in treated achalasia are unclear.

Objective: To investigate the underlying mechanisms of gastroesophageal reflux and gastroesophageal reflux symptoms in treated achalasia patients.

Study design: A prospective observational study.

Study population: Treated achalasia patients with and without gastroesophageal reflux symptoms visiting the outpatient clinic of the Gastroenterology Department.

Main study parameters/endpoints:

Primary:

- Gastroesophageal reflux episodes

Secondary:

- LES pressure
- Esophagogastric junction distensibility
- Sensitivity to acid perfusion and distension
- Level of esophageal stasis
- Width/diameter of the esophagus.

Nature and extent of the burden and risks associated with participation, benefit and group relatedness: Five different measurements will be performed in two subsequent days. Study subjects will undergo an EndoFLIP, an acid perfusion test, combined high-resolution manometry (HRM) and pH-impedance monitoring and finally a timed barium esophagography will be performed. At the beginning of the study participants will be asked to fill in questionnaires. The measurements are standard procedures that are routinely performed at the motility centre of the Gastroenterology department in the evaluation of achalasia patients. The associated complications are minimal. Study subjects will receive 150 euro for study participation, to cover work day loss. The study will give insight in the mechanisms behind GER-related symptoms which is important for the management of these symptoms and potentially have consequences for the choice of the initial treatment for achalasia.

1. INTRODUCTION AND RATIONALE

Achalasia

Achalasia is a rare motility disorder of the esophagus with an annual incidence of 1 per 100.000 persons. Achalasia is characterised by aperistalsis of the esophageal body and dysrelaxation of the lower esophageal sphincter (LES) caused by progressive destruction and degeneration of the neurons in the myenteric plexus.[1] This leads to subsequent retention of food and saliva in the esophagus, resulting in the typical symptoms of achalasia such as dysphagia, chest pain, regurgitation of undigested food and weight loss.[1] Diagnosis is currently based on typical manometric findings; incomplete relaxation of the LES upon deglutition, absence or simultaneous esophageal contractions and often a hypertensive LES. Due to a lack of knowledge on the cause of the disease, the treatment of achalasia is currently purely palliative and focused on symptom relief. The aim of the treatment is to destroy the occluding function of the esophagogastric junction using endoscopic balloon dilatation, surgical Heller myotomy or per-oral endoscopic myotomy (POEM). The therapeutic success of these three treatments is comparable but all have a risk of complications. The most frequent complication post-treatment is gastroesophageal reflux (GER).[2-4] This is not surprising because the occluding function of the esophagogastric junction is destroyed and thereby the mechanism that protects the esophagus against reflux of gastric content is compromised. The incidence of GER varies and is dependent on the type of treatment; 15%-33% in pneumodilatation, 31.5% in Heller myotomy without fundoplication compared to 8.8%-23% in Heller myotomy with fundoplication.[2,5]

GER in treated achalasia

Achalasia and gastroesophageal reflux disease (GERD) are generally thought to represent opposite ends of the spectrum of esophageal sphincter dysfunction.[6] In achalasia there is dysrelaxation of the LES. But in GERD the LES is hypotonic or undergoes frequent relaxations which lead to an abundant exposure of the esophagus to gastric components.[1] Typical symptoms of GERD are heartburn, chest pain and/or regurgitation. However, these symptoms are also described in patients with achalasia, in both untreated and treated patients.[6-8] In untreated achalasia symptoms of GER would be expected to be rare because the esophageal sphincter is tightly closed.[7] However studies have described variations in LES pressure during the day in achalasia patients and also transient LES relaxations (TLERs) which could lead to the exposure of gastric components in the esophagus and cause reflux symptoms.[9-11]

Ponds FA, *et al. Gut* 2020; 70:30–39. doi: 10.1136/gutjnl-2020-320772

In treated achalasia the esophagogastric junction is destroyed and thereby treated patients seem to be more susceptible for GER. The mechanisms behind the occurrence of GER symptoms in treated achalasia are unclear. It is hypothesized that these symptoms can be caused by true GERD or by retention and fermentation of retained food in the esophagus due to the impaired esophageal peristalsis. Several studies, as mentioned before have described that true GERD can occur in achalasia patients as a result of transient relaxations of the LES (TLESRs).[10-14] Furthermore after treatment it is suggested that true GERD occurs in patients with a lower mean LES pressure post-treatment.[2,7,8] However, other studies contradict these findings. To distinguish GERD from fermentation a 24-hour combined multichannel intraluminal impedance and pH monitoring can be used. With these methods reflux episodes can be discriminated from periods where there is stasis of food.

Previous studies that investigated GER after pneumodilation or Heller myotomy showed that in a substantial proportion of patients there is discordance between GER symptoms and the results of reflux monitoring.[6-8] Furthermore the studies show considerable variability in the incidence of GER symptoms.[2] At this moment it is unclear why there is a discrepancy in the degree and severity of GER symptoms between treated patients. Additionally the exact mechanism behind these symptoms stays indistinct. It could be due to true GERD or fermentation of food. Futhermore, variability in pain sensitivity or in esophageal distensibility could play a role, in which an impaired distensibility could cause more stasis and symptoms. A better insight in the underlying mechanism of GER symptoms in treated achalasia is important because it could influence the choice of the initial treatment.

In conclusion, post-treatment, patients with achalasia often describe reflux-like symptoms. So far there is no convincing evidence that these symptoms are really caused by GERD. Understanding the mechanism behind GER-related symptoms is important for further treatment of these symptoms and could have consequences for the choice of the initial treatment for achalasia.

2. OBJECTIVES

Our hypothesis is that gastroesophageal reflux symptoms in treated patients with achalasia are dependent on retention and fermentation of food due to a reduced clearance by aperistalsis of the esophageal body rather than the result of true acid reflux episodes. We expect a difference in gastroesophageal reflux episodes, LES pressure, sensitivity to acid and esophageal distension in the treated patients with achalasia that experience

Ponds FA, et al. Gut 2020; 70:30–39. doi: 10.1136/gutjnl-2020-320772

gastroesophageal reflux symptoms compared to the group that does not encounter gastroesophageal reflux symptoms.

Based on the hypothesis the following study objective is formulated.
Primary objective:

- To investigate the underlying mechanisms of gastroesophageal reflux and gastroesophageal reflux symptoms in treated achalasia patients.

3. STUDY DESIGN

This observational study has a prospective design. Two groups of study subjects will be included. Treated patients with achalasia with gastroesophageal reflux symptoms and treated patients with achalasia without gastroesophageal reflux symptoms. On baseline, clinical data will be collected regarding medical history, family history and medical symptoms related to GER or achalasia.

In all the included study subjects five different measurements will be performed in two days, as outlined in the schematic overview under subheading 5.2.1 ‘research protocol’. The measurements will take place in the AMC at the outpatient clinic of the Motility Centre of the Gastroenterology Department. The duration of the study is dependent on the rate of inclusion of the study subjects but, it is expected that in two years all study subjects will be included.

4. STUDY POPULATION

4.1 Population (base) and study enrolment

Patients that visit the outpatient clinic of the Motility Centre of the Gastroenterology Department of the AMC will be approached by the physician of the patient. In case patients are interested to participate the coordinating investigators of the study will provide further information about the study.
Patients should be diagnosed with achalasia by manometry using the predefined manometric criteria and all underwent previous treatment for achalasia. Eligible patients will be given a verbal explanation of the study. Each patient will receive a patient information brochure about the study and an informed consent form for participation. Patients will be given sufficient time to read the information and ask questions. Before any study procedures are initiated the patient must sign the informed consent form.

The annual incidence of achalasia is 1 per 100.000 persons. In the Netherlands a substantial proportion of the patients with achalasia are followed at the Motility Centre of the AMC. The number of patients visiting our department is sufficiently high to find participants for the study.

4.2 Inclusion criteria

4.2.1 Treated achalasia patients with gastroesophageal reflux symptoms

- Diagnosis of idiopathic achalasia confirmed by esophageal manometry that shows the following criteria:
    - Aperistalsis or simultaneous contractions in the esophageal body.
    - LES dysrelaxation.
- Treatment of achalasia with one of the following procedures:
    - Endoscopic balloon dilatation
    - Surgical Heller myotomy
    - per-oral endoscopic myotomy (POEM)
- Minimum total score on the GastroEsophageal Reflux Disease Questionnaire (GERDQ) of ≥ 8.
- Gastroesophageal symptoms after treatment lasting more than 3 months.
- Age 18-80 years.
- Written informed consent.

4.2.2 Treated achalasia patients without gastroesophageal reflux symptoms

- Diagnosis of idiopathic achalasia confirmed by esophageal manometry that shows the following criteria:
    - Aperistalsis or simultaneous contractions in the oesophageal body.
    - LES dysrelaxation.
- Treatment of achalasia with one of the following procedures:
    - Endoscopic balloon dilatation
    - Surgical Heller myotomy
    - per-oral endoscopic myotomy (POEM)
- Maximum total score on the GastroEsophageal Reflux Disease Questionnaire (GERDQ) of < 8.
- No gastroesophageal symptoms after treatment.
- Age 18-80 years.
- Written informed consent.

Ponds FA, *et al. Gut* 2020; 70:30–39. doi: 10.1136/gutjnl-2020-320772

4.3 Exclusion criteria

4.3.1 Treated achalasia patients with gastroesophageal reflux symptoms

- Pseudoachalasia.
- Upper gastrointestinal malignancy.
- Chagas disease.
- Peptic ulcer disease.
- Inability to stop PPI, H2-receptor antagonist or prokinetic drug for two weeks
- Presence of an extremely dilated oesophagus body >5 cm

4.3.2 Treated achalasia patients without gastroesophageal reflux symptoms

- Similar to 4.3.1

4.4 Sample size

Treated achalasia patients with or without gastroesophageal reflux symptoms will be included. Per group we aim to include 20 study subjects. In the literature the incidence of GER symptoms in patients with achalasia post-treatment is very variable. Articles describe an incidence that differs between 10% up till 60%. [7,8] This variability in the incidence is probably caused by the different diagnostic approaches in these studies. Furthermore the GER symptoms do not always correlate with a positive pH-impedance monitoring for GERD or fermentation of food.
Based on this we expect that 30% of the patients without GER symptoms will have gastroesophageal reflux episodes caused by GER or fermentation of food. For the patients with GER symptoms we estimate that in 75% gastroesophageal reflux episodes occur caused by GER or fermentation. We consider a 45% difference in gastroesophageal reflux episodes between the two groups as clinically relevant. When, using a two-group chi-square test with a two-sided significance level of 5% and a power of 80%, the sample size required in each group is 19 subjects. Based on this we aim to include 40 study subjects. Below we have calculated the sample sizes based on our main secondary study parameters to show that with a sample size of 40 study subjects it is possible to attain these secondary study parameters.

We believe that the three main secondary study parameters are LES pressure, esophagogastric junction distensibility and the level of esophageal stasis. Several studies have described that true GERD occurs in patients with a lower mean LES pressure post-treatment.[2,7,8] However, other studies contradict these findings. Based on our hypothesis we believe that patients with GER symptoms have a higher mean LES pressure post-treatment

compared to patients without GER symptoms. A higher mean LES pressure could contribute to retention and fermentation of food in the esophagus. In general, treated patients with a mean LES pressure ≤ 10 mmHg are in remission.[15] Therefore we expect that the LES pressure in patients without GER symptoms is ≤ 10 mmHg and in patients with GER symptoms > 10 mmHg. We consider a difference of 5 mmHg in LES pressure as clinically relevant. When, using a two-group t-test with a two-sided significance level of 5% and a power of 80%, the sample size required in each group is 18 study subjects. For this calculation we assumed that the LES pressure in patients without GER symptoms is 10 mmHg or lower and for patients with GER symptoms we assumed a LES pressure of 15 mmHg or higher. The common standard deviation was assessed at 5.1 mmHg for both groups.

Previous studies did not investigate the role of esophagogastric junction distensibility and esophageal stasis in treated achalasia patients with GER symptoms. Therefore for these parameters, we estimated the difference between the two groups that in our opinion could be clinically relevant. Our hypothesis is that GER symptoms are dependent on retention and fermentation of food due to a reduced clearance by aperistalsis of the esophageal body. Consequently, we expect that the esophagogastric junction (EGJ) distensibility in the group with GER symptoms is decreased and esophageal stasis is increased.
The cut-off for normality of the EGJ distensibility in healthy controls is 2.9 mm^2/mmHg.[16] Values below this cut-off point are considered as a decreased EGJ distensibility. Therefore we expect that in the group without GER symptoms the EGJ distensibility is ≥ 2.9 mm^2/mmHg and in the group with GER symptoms the EGJ distensibility is < 2.9 mm^2/mmHg. A sample size of 12 study subjects in each group will have 80% power to detect a difference in means of at least 1.2 mm^2/mmHg using a two group t-test with a 0.05 two-sided significance level. In this calculation we assumed that patients without GER symptoms have a mean EGJ distensibility of 4.0 mm^2/mmHg and a mean EGJ distensibility of 2.8 mm^2/mmHg or lower is considered as a significant decreased distensibility of the EGJ which is expected in the group with GER symptoms. The common standard deviation was assessed at 1.0 mm^2/mmHg for both groups.
When a barium esophagogram is performed in healthy volunteers there is no stasis of contrast in the esophagus. In achalasia patients, generally there is stasis of contrast during a barium esophagogram, in both treated as untreated patients.[17,18] Stasis of contrast ≥ 5 cm is considered as significant in treated achalasia patients.[17] We expect that in patients with GER symptoms esophageal stasis is increased, ≥ 5cm, compared to patients without GER symptoms. In general all achalasia patients will have some esophageal stasis even without

complaints. Studies have shown that treated patients in remission still have some esophageal stasis, approximately 3.8 cm on the barium esophagogram (rohof). Based on these data we assessed the sample size. A sample size of 17 study subjects in each group will have 80% power to detect a difference in means of at least 1.2 cm using a two group t-test with a 0.05 two-sided significance level. This calculation is based on the assumption that patients without GER symptoms have on the barium esophagogram stasis of around 3.8 cm and stasis of 5 cm or higher is considered as a significant stasis which is expected in patients with GER symptoms. The common standard deviation was assessed at 1.2 cm for both groups.

In the Netherlands a large part of the patients with achalasia are referred to the Motility Centre of the AMC for diagnosis and treatment. Therefore we believe that the number of patients visiting our department is sufficiently high to find enough participants for the study.

5. METHODS

5.1 Study parameters

5.1.1 Primary study parameter

- Gastroesophageal reflux episodes

5.1.2 Secondary study parameter

- LES pressure
- Esophagogastric junction distensibility
- Sensitivity to acid perfusion and distensibility
- Level of esophageal stasis
- Width/diameter of the esophagus

5.2 Study procedures

Treated patients with idiopathic achalasia visiting the outpatient clinic will be asked to participate in the study. Only when a study subject has given informed consent, questionnaires concerning demographic and clinical data will be recorded and measurements will be performed.

5.2.1 Research protocol

Baseline clinical data will be collected using different questionnaires. Before any measurement will be performed the participant will be subjected to the GERD Questionnaire to be certain that he or she is allocated in the correct study group.
For this study all included study subjects will undergo five different measurements in two following days, as outlined in the schematic overview. The first measurement will be performed with impedance planimetry, using the Endo Functional Luminal Imaging Probe (EndoFLIP). The Endoflip can determine the esophagogastric junction distensibility and examine the esophageal distensibility.[16,19] The measurement with the Endoflip will be followed by an acid perfusion test, the Bernstein test. The perfused catheter used for the high-resolution manometry will be used to infuse the acid and control solutions. The acid perfusion test will provide information about the sensitivity for acid in the esophagus. After the acid perfusion test a combined measurement of high-resolution manometry (HRM) and pH-impedance monitoring will be carried out. The catheter used for the acid perfusion test is the same as the catheter for the HRM. After 3-4 hours the HRM catheter will be removed and the pH-impedance catheter will remain in place for a further 24-hour measurement of reflux episodes and symptoms. After 24-hour pH-impedance monitoring the catheter will be removed and a timed barium esophagography will be performed. The barium esophagography is performed to gain insight into esophageal emptying and the width of the esophagus.

Schematic view of the study design

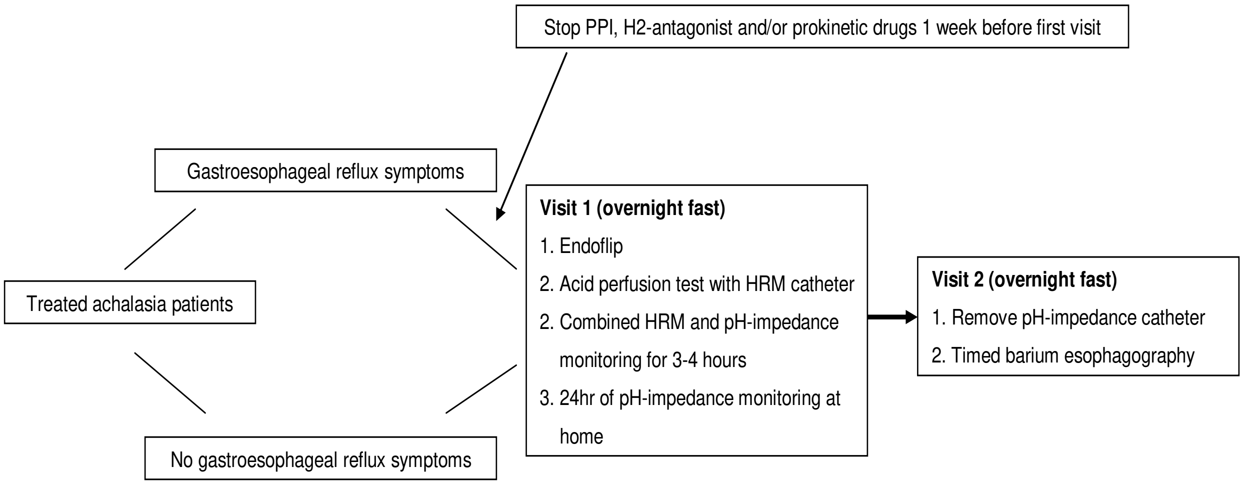

Study requirements and timeline

The measurements will be performed after cessation of PPIs, H2-antagonists and medications which can influence intestinal motility for 1 week. A temporary stop of PPI, H2-antagonist or prokinetic drug prior to reflux monitoring is not harmful for the patient and is in concordance with the reflux guideline of the Dutch association of gastroenterologists. For the reduction of severe symptoms patients are allowed to take rescue medication in the form of antacids. Furthermore, one day before the measurements the patients will be restricted to a liquid diet to minimise stasis of food in the esophagus.

Study subjects will arrive in the morning at the clinic after an overnight fast, no intake of fluid after midnight except water. Preferably the intake of medication should be postponed after the measurements. If this is not possible medication should of course be ingested prior to the measurements. Before the first measurements patients must have been fasting for two hours, so also no intake of water. The catheter for the Endoflip will be placed transnasally. After this measurement the catheters for the esophageal HRM and pH-impedance will be placed transnasally. Before placement of the pH-impedance catheter, an acid perfusion test will be performed using the HRM catheter. The combined HRM and pH-impedance measurement will be performed in the fasting condition for half an hour followed by a postprandial measurement of 3.5 hours after a standardized liquid meal, 400 mL nutrient drink (600 kcal Nutridrink, Nutricia, the Netherlands) with 200 mL water. The liquid meal will be administered by the catheter used for the HRM. After this the HRM catheter will be removed while the pH-impedance catheter will stay in place and reflux will be monitored for the next 20 hours. The standard investigation for patients with reflux symptoms is 24-hour pH-impedance monitoring. Study subjects go home with the pH-impedance catheter in place and will receive a diary to write down there symptoms. During this measurement the study subject has to drink and eat at regular moments but there is no restriction concerning the food intake, except again an overnight fast due the timed barium esophagography the next day. The following day patients come back to the clinic where the pH-impedance catheter will be removed and a timed barium esophagography will be performed, which is the last measurement.

5.2.2 Procedures and questionnaires

Endo Functional Luminal Imaging Probe (EndoFLIP)

To determine the distensibility of the esophagogastric junction (EGJ) and the esophagus the EndoFLIP system will be used (MMS, Enschede, the Netherlands). The measurement with the EndoFLIP will be performed according to a previously described protocol.[16] A probe is

inserted into the esophagus and placed at the level of the EGJ. The probe consists of a 240-cm catheter with a 14-cm bag attached to its distal end, which is compliant to a maximal diameter of 25 mm. Inside the bag 17 electrodes are placed at 4-mm intervals. An excitation current of 100μA is generated between two adjacent electrodes at a frequency of 5 kHz. Using impedance-planimetry cross sectional areas (CSAs) are determined for the 16 balloon cross sections during volume controlled distensions. Additionally two pressure sensors are located on the probe to determine intrabag pressure allowing assessment of EGJ distensibility.

The distension probe and the pressure transducers are calibrated by the manufacturer. The pressure sensor is zeroed prior to transnasally insertion of the catheter. Previous manometry readings of the study subjects will be used to position the balloon at the EGJ. The balloon will be inflated to a 20, 30, 40, 50, 60 and 70 mL volume. Pressures and CSAs are collected at a rate of 10 Hz. Distensibility is assessed using the median value over a 30 s dynamic measurement of the narrowest CSA, which corresponds to the EGJ, and the median intrabag pressure.[19]

After measuring the EGJ distensibility the catheter will be placed 10 cm above the LES and the balloon will be inflated to a 20, 30, 40, 50, 60 and 70 mL volume. The time between each inflation will be 30 seconds and study subjects will be asked after each inflation whether they experience pain or other symptoms.

High Resolution Manometry (HRM)

A water-perfused high-resolution manometry assembly with 21 water-perfused sensors spaced at 1-cm intervals at the position of the esophagogastric junction will be used (Medical Measurement Systems, Enschede, The Netherlands). Each sensor is accurate to within 1mmHg, capable of recording transient pressure changes in excess of 6000 mmHg/s, and zeroed to atmospheric pressure. The high-resolution manometry assembly will be introduced transnasaly and positioned to record from hypopharynx to stomach with about five intragastric sensors. Data are analyzed using Solar high-resolution manometry software (Medical Measurement Systems, Enschede, The Netherlands).

pH- impedance monitoring

A pH-impedance catheter will be introduced transnasally. A 2.1-mm outer diameter study catheter comprised of six electrode pairs measuring intraluminal impedance at 3, 5, 7, 9, 15 and 17 cm above the LES and an ISFET pH sensor, placed 5 cm above the LES will be used. Before and after recording the pH electrode will be calibrated using standard buffers of

pH 7 and pH 1. The catheter will be positioned in such a way that the pH sensor is located 5 cm above the LES, which is based on manometry readings.

Analysis of the data of the HRM and pH-impedance monitoring

For analysis of the data obtained with HRM and pH-impedance measurement MMS software (Enschede, The Netherlands) will be used. Observers will be blinded for patient characteristics when analyzing the measurement.

The HRM will give insight in the esophageal function. It provides information on the contractile activity of the esophageal body and the esophagogastric junction including the LES. Therefore it is helpful in distinguishing transient LES relaxation (TLESRs), which occurs in GERD, from pseudorelaxation of the LES by shortening of the esophageal body. pH-impedance monitoring provides information on esopheageal bolus transit. Combined with HRM it can distinguish GER from retention and fermentation of food. GER is characterized by TLESRs and a retrograde moving decrease in impedance with an abrupt drop in pH levels followed by normalisation of the impedance and pH. In case of fermentation there is a slow and longer decrease in impedance and pH. The pH-impedance monitoring will also record all reflux episodes, both acid as non-acid, during the 24-hours measurement. During the analysis of the data of the pH-impedance monitoring reflux symptoms will be correlated with acid and non-acid reflux episodes. A positive correlation is declared if a specific reflux symptom is preceded by a reflux episode within two minutes.

Acid perfusion test

Patients will undergo an acid perfusion test, according to a previously described protocol.[20] The manometry catheter used for the HRM (Unisensor AG, Attikon Switzerland) will be placed in the esophagus in such as way that the additional side-hole is placed at 5 cm above the LES. After an adaption period of 10 minutes saline will be infused in the distal esophagus for 10 min, followed by hydrochloric acid (0.1N) for 30 min or until the study subject experiences pain. Both saline and hydrochloric acid will be instilled at a rate of 8 mL/min, controlled by an automatic pump (IVAC 560 Volumetric Pump; Rhys Int. Ltd, Bolton, UK). Study subjects will be blinded to the nature of the infusate and will be asked to report the first sensation of heartburn, discomfort and pain. When study subjects experience pain, acid infusion will be discontinued immediately. After every elapsed minute during the acid perfusion test, patients will be asked whether their sensation changes. The time from start of acid infusion (marked by a pH drop to pH < 1.0), necessary to provoke first perception, discomfort and pain will be noted and represents a measure of sensitivity.

Timed barium esophagram

A maximal tolerable amount of low density barium sulphate has to be ingested by the study subjects in 30-45 s without regurgitation or aspiration. After ingestion of low density barium sulphate esophageal stasis will be determined at 1, 2 and 5 minutes with the study subject upright in a slight left posterior position. The distance from the tapered distal esophagus to the top of the barium column and the maximal diameter of the esophagus are measured.

Questionnaires

- Eckardt score, which is the sum of symptom scores for dysphagia, regurgitation, chest pain an weight loss. Each symptom is scored form 0 to 3. The minimum score is 0 and the maximum score is 12.
- The validated Achalasia Disease-Specific Quality-of-Life questionnaire (Achalasia-DSQoL).[21]
- The validated Gastroesophageal Reflux Disease Questionnaire (GERDQ) is a self-assessment questionnaire that can be used to for the diagnosis and follow-up of gastroesophageal reflux disease. It measures both symptoms and impact of symptoms on person’s daily life.
- Medical Outcomes Study 36-Item Short-Form Health Survey (SF-36). The SF-36 mental and physical summary scores (range from 0-100 and higher scores indicate better well-being) measure general aspects of health quality of life.[21,22]

5.3 Withdrawal of individual subjects

Participants can leave the study at any time for any reason if they wish to do so without any consequences. The investigator can decide to withdraw a participant from the study for urgent medical reasons. In case of withdrawal all stored data and results of previously analysis will not be destroyed expect when the participant asks for destruction of the data. Consequently, the results of the data will also be destroyed.

6. SAFETY

6.1 Section 10 WMO event

In accordance to section 10, subsection 1, of the WMO, the investigator will inform the subjects and the reviewing accredited METC if anything occurs, on the basis of which it appears that the disadvantages of participation may be significantly greater than was foreseen in the research proposal. The study will be suspended pending further review by

the accredited METC, except insofar as suspension would jeopardise the subjects' health. The investigator will take care that all subjects are kept informed.

6.2 Adverse events and serious adverse events

All the measurements that are performed are safe procedures and routinely performed in the clinical setting, with rarely any serious complications. Possible complications are mainly due to placement of the catheters. The catheters can give discomfort in the nose and pharynx. Furthermore in rare cases a mucosal bleeding of the nose, caused by the catheter, can occur which never need extra treatment. All adverse events reported spontaneously by the subject or observed by the investigators will be recorded in a database.

All serious adverse events (SAEs) will be reported through the web portal *ToetsingOnline* to the accredited METC that approved the protocol. The SAEs will be reported within 15 days after the sponsor is first notified of the SAEs.
The SAEs that result in death or are life treating will be reported expedited through the web portal *ToetsingOnline* to the accredited METC that approved the protocol. This will not occur later than 7 days after the coordinating investigators and principal investigator have knowledge of the SAE(s). This first report is preliminary and within 8 days after submission of the first report a final report will be submitted.

6.3 Follow-up of adverse events

All adverse events will be followed until they have abated or until a stable situation has been reached. Depending on the event, follow up may require additional tests or medical procedures as indicated, and/or referral to another medical specialist.

6.4 Annual safety report

Not applicable for this study.

7. PRIVACY

The data of the subjects are coded in order of participation. This code will identify participants throughout documentation and evaluation. The code does not obtain elements of the name or date of birth of the participants. The code and the data are stored in different locations. The code can only be seen by the investigators. The participants will be told that all study findings will be stored on the computer and handled in strict confidence according to national guidelines. Study data will be stored 20 years after closure of the study.

Ponds FA, *et al*. *Gut* 2020; 70:30–39. doi: 10.1136/gutjnl-2020-320772

8. STATISTICAL ANALYSIS

Statistical analysis will be performed in collaboration with the department of Biostatistics and Epidemiology of the University of Amsterdam. After testing for normality, unpaired testing will be performed between the two treatment groups for all primary and secondary outcome parameters. Categorical variables will be compared using the Chi-square test. Comparison of the numerical variables will be dependent of the distribution of the data.

9. ETHICAL CONSIDERATIONS

9.1 Regulation statement

The procedures set out in this study protocol are designed to ensure that the investigators abide by the principles of the good clinical practice guidelines of the European Union and the Declaration of Helsinki in the conduct, evaluation and documentation of the study. The protocol of this study will be submitted to the Medical Ethical Committee of the Academic Medical Centre and will not start before formal approval has been granted.

9.2 Recruitment and consent

Participants will be given oral explanation about the study and receive an information letter including an informed consent form. Patients will be given sufficient time to read the information and ask questions. Before any study procedures are initiated the patient must sign the informed consent form. Participants are allowed to withdraw informed consent without providing arguments.
Patients with achalasia with or without gastroesophageal reflux symptoms are suitable candidates for the study and will be recruited from the outpatient clinic. Patients that fulfill the criteria are asked to participate in the study.

9.3 Benefits and risks assessment

Benefits and risks of study participation are both nearly absent. The study can provide more insight in the mechanism behind GER symptoms in treated achalasia patients. Therefore it could be possible to improve the treatment of these patients and the results might even influence the choice of the initial treatment of achalasia.

9.4 Compensation for injury

Ponds FA, *et al. Gut* 2020; 70:30–39. doi: 10.1136/gutjnl-2020-320772

The investigator has a liability insurance which is in accordance with article 7, subsection 9 of the WMO.

This insurance provides cover for damage to research subjects through injury or death caused by the study.

1. € 450.000,-- (i.e. four hundred and fifty thousand Euro) for death or injury for each subject who participates in the Research;
2. € 3.500.000,-- (i.e. three million five hundred thousand Euro) for death or injury for all subjects who participate in the Research;
3. € 5.000.000,-- (i.e. five million Euro) for the total damage incurred by the organisation for all damage disclosed by scientific research for the Sponsor as 'verrichter' in the meaning of said Act in each year of insurance coverage.

The insurance applies to the damage that becomes apparent during the study or within 4 years after the end of the study.

9.5 Incentives

There is an incentive of 150 euro per study subject for participation in the project and compensation for travel expensions. Regular treatment by the local Gastroenterologist is not influenced by participation or refusal.

10. ADMINISTRATIVE ASPECTS AND PUBLICATION

10.1 Handling and storage of data and documents

All the stored data and documents will be coded with a study number, in order of participation, and the date of inclusion. The code is unique for every participant that contributes to this study and does not obtain elements of the name or date of birth of the study subjects. This code will identify study subjects throughout documentation and evaluation. The code of the data of the participants is stored in different locations. Therefore, this code will not be directly traceable to the participant. Only the involved investigators will have access to the code. The participants will be told that all study findings will be stored on the computer and handled in strict confidence according to national guidelines.

The principal investigator and the main investigator will have access to the code and thedata. Auditors, monitors and the Health Care Inspectorate will have access to the data. The data will not be available to other reachers. After closure of the study, the study data will be stored for 20 years.

Ponds FA, *et al. Gut* 2020; 70:30–39. doi: 10.1136/gutjnl-2020-320772

Study subjects can only be included in the study when written informed consent is given. During the study, participants can withdraw consent at any time for any reason without any consequences. By withdrawal of the study no additional information of the participants will be collected. The stored data and results of previously analysis will not be destroyed if a study subject withdrawals. But participants keep the right to ask for destruction of the data and consequently the results, even after closure of the study. Collecting of extra data or performing more measurements is only allowed when the participant gives a new written informed consent.

10.2 Amendments

Amendments should be made only in exceptional cases once the study has started. Changes must be agreed to in writing, and signed, by all parties concerned. The changes then become part of the study protocol. The medical ethics committee must be informed of all amendments and if necessary approval must be sought for ethical aspects.

10.3 Annual progress report

The investigator will submit a summary of the progress of the study to the accredited METC once a year. Information will be provided on the date of inclusion of the first subject, numbers of subjects included and numbers of subjects that have completed the study, serious adverse events/ serious adverse reactions, other problems, and amendments.

10.4 End of study report

The investigator will notify the accredited METC of the end of the study within a period of 8 weeks. In case the study is ended prematurely, the investigator will notify the accredited METC, including the reasons for the premature termination. Within one year after the end of the study, the investigator will submit a final study report with the results of the study, including any publications/abstracts of the study, to the accredited METC.

11. REFERENCES

[1] G. E. Boeckxstaens, "The lower oesophageal sphincter," 17 Suppl 1, 13 (2005).

[2] G. M. Campos, et al., "Endoscopic and surgical treatments for achalasia: a systematic review and meta-analysis," 249(1), 45 (2009).

[3] A. Csendes, et al., "Very late results of esophagomyotomy for patients with achalasia: clinical, endoscopic, histologic, manometric, and acid reflux studies in 67 patients for a mean follow-up of 190 months," 243(2), 196 (2006).

Supplemental material BMJ Publishing Group Limited (BMJ) disclaims all liability and responsibility arising from any reliance placed on this supplemental material which has been supplied by the author(s) *Gut*

[4] S. R. Lopushinsky and D. R. Urbach, "Pneumatic dilatation and surgical myotomy for achalasia," 296(18), 2227 (2006).

[5] G. E. Boeckxstaens, et al., "Pneumatic dilation versus laparoscopic Heller's myotomy for idiopathic achalasia," 364(19), 1807 (2011).

[6] P. F. Crookes, S. Corkill, and T. R. DeMeester, "Gastroesophageal reflux in achalasia. When is reflux really reflux?," 42(7), 1354 (1997).

[7] S. H. Anderson, et al., "The relationship between gastro-oesophageal reflux symptoms and achalasia," 18(4), 369 (2006).

[8] P. A. Novais and E. M. Lemme, "24-h pH monitoring patterns and clinical response after achalasia treatment with pneumatic dilation or laparoscopic Heller myotomy," 32(10), 1257 (2010).

[9] M. A. van Herwaarden, M. Samsom, and A. J. Smout, "Prolonged manometric recordings of oesophagus and lower oesophageal sphincter in achalasia patients," Gut 49(6), 813 (2001).

[10] D. A. Katzka, M. Sidhu, and D. O. Castell, "Hypertensive lower esophageal sphincter pressures and gastroesophageal reflux: an apparent paradox that is not unusual," 90(2), 280 (1995).

[11] J. P. Shoenut*, et al.*, "Reflux in untreated achalasia patients," 20(1), 6 (1995).

[12] J. P. Shoenut, D. Duerksen, and C. S. Yaffe, "A prospective assessment of gastroesophageal reflux before and after treatment of achalasia patients: pneumatic dilation versus transthoracic limited myotomy," 92(7), 1109 (1997).

[13] D. Sifrim, J. Janssens, and G. Vantrappen, "Transient lower esophageal sphincter relaxations and esophageal body muscular contractile response in normal humans," Gastroenterology 110(3), 659 (1996).

[14] S. J. Spechler*, et al.*, "Heartburn in patients with achalasia," Gut 37(3), 305 (1995).

[15] V. F. Eckardt, C. Aignherr, and G. Bernhard, "Predictors of outcome in patients with achalasia treated by pneumatic dilation," Gastroenterology 103(6), 1732 (1992).

[16] W. O. Rohof*, et al.*, "Efficacy of treatment for patients with achalasia depends on the distensibility of the esophagogastric junction," Gastroenterology 143(2), 328 (2012).

[17] W. O. Rohof, A. Lei, and G. E. Boeckxstaens, "Esophageal stasis on a timed barium esophagogram predicts recurrent symptoms in patients with long-standing achalasia," 108(1), 49 (2013).

[18] M. F. Vaezi*, et al.*, "Timed barium oesophagram: better predictor of long term success after pneumatic dilation in achalasia than symptom assessment," Gut 50(6), 765 (2002).

[19] M. A. Kwiatek*, et al.*, "Esophagogastric junction distensibility after fundoplication assessed with a novel functional luminal imaging probe," 14(2), 268 (2010).

[20] G. J. Hemmink*, et al.*, "Does acute psychological stress increase perception of oesophageal acid?," 21(10), 1055 (2009).

[21] R. Frankhuisen, *et al.*, "Validation of a disease-specific quality-of-life questionnaire in a large sample of Dutch achalasia patients," 21(6), 544 (2008).

[22] J. E. Brazier, *et al.*, "Validating the SF-36 health survey questionnaire: new outcome measure for primary care," 305(6846), 160 (1992).
